# Abundance of metalloprotease FtsH12 modulates chloroplast development in *Arabidopsis thaliana*

**DOI:** 10.1093/jxb/eraa550

**Published:** 2020-11-20

**Authors:** Kati Mielke, Raik Wagner, Laxmi S Mishra, Fatih Demir, Andreas Perrar, Pitter F Huesgen, Christiane Funk

**Affiliations:** 1 Department of Chemistry, Umeå University, Umeå, Sweden; 2 Central Institute for Engineering, Electronics and Analytics, Jülich, Germany; 3 CECAD, Medical Faculty and University Hospital, University of Cologne, Cologne, Germany; 4 Institute of Biochemistry, University of Cologne, Cologne, Germany; 5 Swedish University of Agricultural Sciences, Sweden

**Keywords:** *Arabidopsis thaliana*, chloroplast, degradomics, FtsH metalloprotease, protein import, proteomics

## Abstract

The ATP-dependent metalloprotease FtsH12 (filamentation temperature sensitive protein H 12) has been suggested to participate in a heteromeric motor complex, driving protein translocation into the chloroplast. FtsH12 was immuno-detected in proplastids, seedlings, leaves, and roots. Expression of Myc-tagged FtsH12 under its native promotor allowed identification of FtsHi1, 2, 4, and 5, and plastidic NAD-malate dehydrogenase, five of the six interaction partners in the suggested import motor complex. *Arabidopsis thaliana* mutant seedlings with reduced *FTSH12* abundance exhibited pale cotyledons and small, deformed chloroplasts with altered thylakoid structure. Mature plants retained these chloroplast defects, resulting in slightly variegated leaves and lower chlorophyll content. Label-free proteomics revealed strong changes in the proteome composition of *FTSH12* knock-down seedlings, reflecting impaired plastid development. The composition of the translocon on the inner chloroplast membrane (TIC) protein import complex was altered, with coordinated reduction of the FtsH12-FtsHi complex subunits and accumulation of the 1 MDa TIC complex subunits TIC56, TIC214 and TIC22-III. *FTSH12* overexpressor lines showed no obvious phenotype, but still displayed distinct differences in their proteome. N-terminome analyses further demonstrated normal proteolytic maturation of plastid-imported proteins irrespective of *FTSH12* abundance. Together, our data suggest that FtsH12 has highest impact during seedling development; its abundance alters the plastid import machinery and impairs chloroplast development.

## Teaser

Modulation of metalloprotease FtsH12 abundance in Arabidopsis results in altered composition of the plastid import machinery, which in turn affects the development of functional chloroplasts.

## Introduction

Proteolysis is a degradative process that not only supplies nutrients, controls protein amounts or removes damaged or superfluous proteins, but also allows post-translational protein modifications and signaling (Turk *et al.*, 2012; Salvesen *et al.*, 2016). FtsH (filamentation temperature sensitive protein H) proteases are a family of membrane-bound metalloproteases present in eubacteria, animals, and plants. These indispensable members of the M41 peptidase family (Rawlings *et al.*, 2017) consist of an AAA (ATPase associated with various cellular activities) domain and a metalloprotease domain ligating Zn^2+^ in the consensus sequence HEXXH (where X is any uncharged residue). They are inserted into the biological membrane with one or more transmembrane helices located at the N-terminus of the enzyme (reviewed in Wagner *et al.*, 2012; Kato and Sakamoto, 2018). The AAA-type domain is involved in the formation of hexameric complexes, provides ATPase function to extract membrane substrates, and opens and closes the C-terminal hexameric cage formed by the M41 peptidase domains (Okuno and Ogura, 2013).

Compared with non-photosynthetic organisms, plants and cyanobacteria contain more *FTSH* genes, with 17 members present in the model plant *Arabidopsis thaliana* (Wagner *et al.*, 2012). Twelve of these enzymes contain the typical zinc-binding motif required for proteolytic activity, while five carry mutations in the HEXXH motif, which render them presumably proteolytically inactive; they were therefore termed FtsHi. The sub-cellular localization of eukaryotic FtsH proteins is restricted to organelles of endosymbiotic origin (mitochondria and chloroplasts). Sub-cellular fractionation in combination with immunoblot and mass spectrometric analysis identified five of the 13 plastid-localized FtsH enzymes in the thylakoid membrane, the photosynthetic membrane of the chloroplasts. Four of those FtsHs, FtsH1, 2, 5, and 8, are highly expressed and form well-studied heteromeric complexes important for the degradation and assembly of D1 protein and other transmembrane subunits of the photosynthetic machinery (reviewed in Putarjunan *et al.*, 2013; Kato and Sakamoto, 2018). The thylakoid membrane-localized FtsH6 was recently shown to be involved in thermo-memory of seedlings of Arabidopsis (Sedaghatmehr *et al.*, 2016), while adult plants did not show any phenotype when grown in semi-natural outdoor conditions (Wagner *et al.*, 2011). The remaining four plastid-located FtsH proteases (FtsH7, 9, 11, and 12), as well as the five presumably proteolytically inactive FtsHi enzymes, were observed in the plastid envelope using proteomics approaches (Ferro *et al.*, 2010). While the location of FtsH11 (Wagner *et al.*, 2016) and FtsH12 (Li *et al.*, 2017) in the inner envelope could be confirmed, an unexpected localization of FtsHi4 in the thylakoid membrane has been reported (Lu *et al.*, 2014). With the exception of *FTSHi*3, *FTSHi* knock-out mutants are embryo lethal (Meinke *et al.*, 2008), with reduced expression showing diminished Darwinian fitness (Mishra *et al.*, 2019). A point mutation in *FTSHi1* that impaired its ATPase domain function (*ftsh1-1/arc1;*Kadirjan-Kalbach *et al.*, 2012) as well as knock-down mutants of *FTSHi4* (Lu *et al.*, 2014) and *FTSHi5* (Wang *et al.*, 2018) showed cotyledon and growth phenotypes, including alterations in chloroplast ultrastructure. Plants lacking the active envelope-located protease FtsH11 were found to be sensitive to continuous light (Wagner *et al.*, 2011) and high temperature (Chen *et al.*, 2016; Adam *et al.*, 2019). FtsH12 is the largest member of the presumably proteolytically active FtsH family in *A. thaliana*. *FtsH12* knock-out results in developmental arrest of the embryo (Meinke *et al.*, 2008).

Recent immunoprecipitation studies using transgenic lines overexpressing *FTSH12* showed that FtsH12 forms a heteromeric 2 MDa complex with FtsHi1, FtsHi2, FtsHi4, and FtsHi5, the plastidic NAD-dependent malate dehydrogenase (pdNAD-MDH) and hypothetical chloroplast open reading frame (YCF) 2 (Schreier *et al.*, 2018). It was further reported that this heteromeric FtsH12-FtsHi complex associates with the plastid import complex formed by the subunits 20, 56, 100, 214 (YCF1) of the translocon at the inner chloroplast envelope membrane (TIC), and drives protein import with a pulling force generated by its ATPase subunits (Kikuchi *et al.*, 2018). Interestingly, knock-out mutants of any of the subunits of the FtsH12 motor complex are embryo-lethal, whereas single knock-out mutants of TIC20, TIC56 and TIC100 develop albino seedlings (Nakai, 2018). FtsH12 was thus suggested to be an essential component of an ATP-driven motor complex, while its proteolytic activity was reported to be dispensable (Kikuchi *et al.*, 2013). However, neither FtsH12 orthologues nor FtsHi are found in *Poaceae* (Nakai, 2018; Mishra *et al.*, 2019; Li *et al.*, 2020), suggesting that it is not ubiquitous in all higher plants. Therefore, the importance of both the FtsH12-FtsHi complex and the interacting 1 MDa TIC complex for plastid protein import are subjects of a controversial debate (Nakai, 2015; Agne *et al.*, 2017; Bölter and Soll, 2017; Li *et al.*, 2020; Nakai, 2020). The consequences of modulated FtsH12 expression have not been investigated so far.

In this study, we characterized the expression and topology of FtsH12 as well as the phenotypes, morphology, proteomes, and N-terminomes of *A. thaliana* lines with modulated expression of *FTSH12*. Sub-cellular fractionation and *in organello* protease protection assays confirmed localization of FtsH12 in the inner envelope of the chloroplast. Diminished amounts of FtsH12 in the chloroplasts had a strong impact on the plant phenotype and plastid morphology. *FTSH12* expression was not only observed in chloroplasts, but even in plastids of roots and in proplastids. Expression of Myc-tagged FtsH12 under its native promoter confirmed FtsHi1, FtsHi2, FtsHi5, and plastid NAD-MDH as interaction partners in a heterooligomeric FtsH12 complex. Seedlings with increased or reduced FtsH12 expression revealed strong changes in the proteome, including the components of the plastid protein import machinery. While the abundance of the FtsH12-FtsHi complex was diminished, TIC22-III protein accumulated in plants with reduced amounts of FtsH12. N-terminome analysis further demonstrated normal proteolytic plastid protein maturation in plants with altered *FTSH12* expression. This suggests that the delayed chloroplast development in FtsH12-depleted plants was caused by limited plastid protein import capacity, which is particularly important in the early growth stages.

## Materials and methods

### Plant material and growth conditions

*A. thaliana* ecotype *Columbia-0* (Col-0) was used as wild type control and as background line to generate *FTSH12* miRNA knock-down mutants. A GABI-Kat T-DNA insertion line (GABI_550G09; Kleinboelting *et al.*, 2012) was used as background line for expression of C-terminal Myc-tagged FtsH12 under its native promoter (Supplementary Table S1). Seeds were plated on Murashige and Skoog (MS; Murashige and Skoog, 1962) medium and when needed, supplemented with 50 µg ml^-1^ antibiotics. After stratification at 4 °C plants were grown in short day (8 h/ 16 h photoperiod, 22 °C/ 18 °C, 120 μmol photons m^−2^ s^−1^). After two weeks, plants were transferred to soil and kept at short day conditions. Phenotypes of seedlings and leaves were documented using a Leica MZ9.5 microscope (Germany) or Epson Perfection 3200 PHOTOscanner (Japan). Tip-to-tip distance of cotyledons of two-day-old seedlings, as well as the rosette diameter of seven-week-old plants were measured using ImageJ (https://imagej.nih.gov/ij/). For etiolated seedlings, germination was stimulated by placing the seeds on plates under white light (150 μmol photons m^−2^ s^−1^) for 3–5 h, followed by growth for 5–6 d in the dark at 22 °C. The dark-grown seedlings at 0 h were harvested under low green light illumination. To identify inactive FtsHi enzymes of Arabidopsis, mutants were screened for homozygous knock-outs from a collection of heterozygous T-DNA insertion lines (Mishra *et al.*, 2019). The T-DNA insertion lines used were as follows: FtsHi1 (AT4G23940) ARC1; FtsHi2 (AT3G16290), *ftshi2-5* (SAIL_1178_A11); FtsHi4 (AT5G64580), *ftshi4-1* (GK-382B06) and *ftshi4-2* (SALK_067969.20.10.x); and FtsHi5 (AT3G04340), *ftshi5-1* (GK-058A04).

### Plasmid construction and plant transformation

Knock-down *FTSH12* lines (*mi12*) were generated via micro-RNA constructs according to a previous report (http://wmd3.weigelworld.org/cgi-bin/webapp.cgi; Schwab *et al.*, 2006). With specifically designed primers (‘*mi12-1/2/3* I/II/III/IV miR –s/-a/*s/*a’; Supplementary Table S1), two independent regions of *FTSH12* (AT1G79560) were targeted. PCR products were cloned into the plasmid RS300 (MIR319a; Addgene, Watertown USA). cDNA from *A. thaliana* was generated by RevertAid RT Reverse Transcription Kit (Thermo Scientific USA). The coding sequence of *FTSH12* was synthesized from cDNA with primers ‘ftsh12 Forward’ and ‘ftsh12 Reverse for overexpressor’ (Supplementary Table S1) and cloned into the binary vector pk2GW7 (VIB, Gent, Belgium) under a 35S promoter. For GUS staining, the natural promotor of *FTSH12* (‘ftsh12 Promotor Forward’, ‘ftsh12 Promoter Reverse’, Supplementary Table S1) from *A. thaliana* gDNA was cloned into the destination vector pBGGUS (Kubo *et al.*, 2005) by fusing it with the *GUS* reporter gene. A fusion construct was synthesized by overlapping PCR using the amplified *FTSH12* promoter and CDS as template and the primers ‘ftsh12 Promoter Forward’ and ‘ftsh12 Reverse for cmyc-line’ (Supplementary Table S1). The *pftsh12::ftsh12CDS* was cloned into a pENTR/D-TOPO vector and transferred into the destination vector pGWB16 resulting in a gene product with 4× c-myc tag. Binary plasmids were transformed into electro-competent *Agrobacterium tumefaciens* [GV3101::pMP90 (pTiC58DT-DNA); Hellens *et al.*, 2000]. Non-segregating T2 lines of *A. thaliana* Col-0 were transformed as described by Clough and Bent (1998). Heterozygous T-DNA insertion lines of the GABI-KAT collection (Kleinboelting *et al.*, 2012) were transformed with the T-DNA inserted into *FTSH12* (GABI_550G09). Presence of the c-myc-fused gene product in the T2 generation was confirmed immunologically using anti-FtsH12 antibodies.

### Arabidopsis cell culture

The *A. thaliana* Col-0 cell culture was grown in MS medium supplied with 3% (w/v) sucrose (pH 5.7) at 25 °C in darkness (Pesquet *et al.*, 2010; Dubreuil *et al.*, 2018). Cells were sub-cultured weekly by 1:10 dilution. To immunodetect FtsH12, seven-day-old cells grown in darkness were sub-cultured (1:10 ratio) in MS medium supplied with 1% sucrose and placed in continuous light (150 µmol photons m^-2^ s^-1^). Sampling took place after 0, 1, 5, 7, and 14 d of illumination. Cells were pelleted without centrifugation and snap frozen in liquid nitrogen.

### Histochemical staining of β-glucuronidase (GUS)

Seedlings, leaves, and roots were fixed in 90% acetone at –20 °C for 60 min. Siliques were sliced open before fixation. GUS staining was performed as described by Vielle-Calzada *et al.* (2000). Samples were visualized using a Leica MZ9.5 or Axioplan 2 imaging microscope (Carl Zeiss, Germany).

### Histochemical detection of reactive oxygen species (ROS)

ROS staining was carried out as described by Fryer *et al.* (2002). The experiments were performed on 10-day-old seedlings grown in continuous light for 7 d. ROS staining of plants grown in short day conditions was included as control. To visualize superoxide (O_2_^.-^), the nitroblue tetrazolium (NBT) staining method was used. Hydrogen peroxide (H_2_O_2_)-staining was carried out by using 3,3´-diaminobenzidine (DAB).

### Determination of chlorophyll and anthocyanin content

Chlorophyll extraction was performed on 10-week-old plants grown under short day conditions by grinding the leaves (100 mg) in buffered acetone solution, as described by Porra *et al.* (1989). Anthocyanin extraction was performed on etiolated seedlings harvested at 0 h, day 1, and day 2, as described by Nakata *et al.* (2013).

### Determination of chlorophyll fluorescence parameters

Pulse-Amplitude Modulation (PAM) was measured on seven-week-old plants kept in continuous light for 7 d; plants grown in short day conditions were included as control. All plants were dark adapted for at least 30 min prior to measurements. A PAM-210 fluorimeter (Walz, Germany) was used for measurements over time, with increasing photosynthetic active radiation.

### Chloroplast isolation and sub-fractionation

Chloroplast isolation and sub-fractionation was performed as described by Wagner *et al.* (2016).

### Protease protection assay

*A. thaliana* plants were grown in a light/ dark cycle of 8 h/ 16 h. Chloroplasts were isolated according to Sun *et al.* (2011). The protease protection assay was carried out as described by Knopf *et al.* (2012).

### Protein extraction

Total protein was extracted from 10 day-old seedlings in extraction buffer [50 mM Tris-HCl, pH 7.5, 5 mM EDTA, 10% (v/v) glycerol, 0.5% (w/v) lithium dodecyl sulfate (LDS), 10 mM DTT and protease inhibitor (one tablet per 10 ml buffer; Pierce Thermo Scientific USA) in a mortar pre-cooled with liquid nitrogen. The protein content was quantified using the RC DC Protein Assay Kit (Bio-Rad USA).

### SDS-PAGE and immunoblotting

A total of 15 µg protein per sample was separated on a TGX GEL 4–20 % (Bio-Rad), according to the method described by Laemmli (1970), and transferred to polyvinylidine difluoride (PVDF) membranes (Bio-Rad), as described by Towbin *et al.* (1979). Antigen–antibody complexes were visualized using WesternBright Quantum HRP substrate (Advansta, USA) and the Bio-Rad ChemiDoc™ Imaging Systems (UK).

Immunoblotting was performed as described by Wagner *et al.* (2016). A polyclonal antibody directed against the C-terminal FtsH12 peptide sequence DRVSYQPVDLRAAPLHRS was raised in rabbit and was produced at Agrisera, Sweden. Antibodies against CP43 and TOC75 were purchased from Agrisera AB (Sweden; see Wagner *et al.*, 2016).

### Determination of chloroplast size

Cotyledons of two-day-old seedlings were sliced in isotonic buffer [20 mM Tricine pH 7.8, 0.33 M sorbitol, 5 mM EDTA, 10 mM Na_2_CO_3_, 0.1% (w/v) BSA]. Released chloroplasts were visualized with an Axioplan 2 light microscope (Carl Zeiss, Germany). The open source image processing program ImageJ (Java-based image processing program developed at the NIH) was used for measuring chloroplast length and width. Average diameters were calculated by dividing the sum of length and width by 2.

### Transmission electron microscopy (TEM)

Transmission electron microscopy was used to study chloroplast morphology in cotyledons of 10-day-old seedlings, and in true leaves of nine-week-old plants of wild type and two *FTSH12* knock-down lines (*mi12-2* and *mi12-3*). In the *FTSH12* knock-down lines, only the morphology of chloroplasts in variegated leaf areas was analyzed. Leaf samples were fixed with 2.5% glutaraldehyde in 0.1 M sodium cacodylate buffer, while the cell suspension was post-fixed in 1% osmium tetroxide, dehydrated with ethanol, propylene oxide and finally embedded in Spurr resin, according to standard procedures (Wagner *et al.*, 2016). Chloroplast length and width were measured by using ImageJ software; average diameters were calculated by dividing the sum of length and width by 2.

### RNA extraction and qRT–PCR

RNA extraction and quantitative real-time polymerase chain reaction (qRT–PCR) was performed as described by Mishra *et al.* (2019).

### Seedling proteome and terminome analysis

Four replicates of seeds from wild type, miRNA *mi12-3*, and *oxp12-1* were grown on MS media plates for two days, with 24 h difference between each replicate sowing. Seedlings were harvested and homogenized in 100 mM HEPES, 5 mM EDTA, 4 M guanidinium chloride (GuHCl), pH 7.5, with 1x Halt protease inhibitors (Thermo Fisher, Germany) using a mortar and pestle. Homogenates were filtered through a 100 µm nylon mesh, centrifuged at 500 × *g* for 1 min at 4 °C, and the filtrate was centrifuged at 10 000 *× g* for 1 min at 4 °C. Proteins in the supernatant were purified by chloroform/methanol precipitation (Wessel and Flügge, 1984) and resolubilized in 6 M GuHCl, 100 mM HEPES pH 7.5. Protein concentration was estimated using the BCA assay (Bio-Rad). For each replicate of each genotype, 100 µg proteome was used for label-free quantitative proteome analysis, and 1 mg proteome for N-terminome analysis. An estimated 100 µg protein of each sample was reduced with 5 mM dithiothreitol for 30 min at 56 °C, cooled to 20 °C and alkylated with 15 mM iodoacetamide for 30 min in darkness, before 1:6 dilution with water, addition of 5 mM CaCl_2_ and 5% (v/v) acetonitrile (ACN). The samples were digested with 1 µg MS-grade approved trypsin (SERVA, Germany) overnight at 37 °C, before addition of a further 0.5 µg trypsin and incubation for 2 h at 37 °C. For terminome analysis, 1 mg wild type, *mi12-3* and *ox12-1* seedlings, proteomes were differentially labeled with stable isotope at the N-terminus and Lys side chain primary amines, using ^12^CH_2_O and sodium cyanoborohydride (for wild type), ^12^CD_2_O and sodium cyanoborohydride (for *mi12-3*), and ^13^CD_2_O and sodium cyanoborodeuteride (for *ox12-1*), as described previously (Demir *et al.*, 2017). Proteins were digested with trypsin and N-termini enriched with a development version of the HUNTER protocol (Weng *et al.*, 2019). Briefly, free trypsin-generated primary amines of internal and C-terminal peptides were modified by hydrophobic tagging with undecanal, the solvent was removed, the pellet resuspended and applied on to a reverse phase solid phase extraction cartridge (SepPak C18, Waters, UK). Dimethylated or endogenously blocked N-terminal peptides were eluted with a stepped gradient from 15–50% (v/v) ACN, whereas hydrophobically tagged, trypsin-generated peptides remained bound to the cartridge under these conditions (Chen *et al.*, 2016).

### Co-immunoprecipitation (Co-IP) of FtsH12-4×c-myc

Chloroplasts of six-week-old *pftsh12::ftsh12CDS::4×c-myc* in GABI_550G09 background were isolated as described above. Plastids were solubilized in PBS, 0.28% beta-DM and 1.86% digitonin. Immunoaffinity enrichment of c-myc tagged FtsH12 and mass spectrometry-based identification of co-precipitating proteins was performed according to Haselmann *et al.* (2018). Antibodies used were monoclonal mouse anti-c-myc and mouse anti-His (Thermo Fisher Scientific). Briefly, proteins eluted after affinity capture were purified with SDS-PAGE and in-gel digested with trypsin (SERVA), as described earlier for proteome analysis by mass spectrometry.

### Mass spectrometry data acquisition

Peptides were de-salted using C18 STAGE-tips (Rappsilber *et al.*, 2007) before analysis with an Ultimate 3000 RSLCnano chromatography system (Thermo, Germany) operated in a two-column setup (Acclaim PepMap100 2 cm trap, 25 cm or 50 cm analytical column). The nanoLC system was linked online to an Impact II high resolution Q-TOF mass spectrometer (Bruker, Germany) via a CaptiveSpray nano ESI source (Bruker), essentially as described previously (Beck *et al.*, 2015). For shotgun proteome analysis, an estimated 1 µg of desalted peptides was loaded, for terminome experiments 50% of each fraction, and for AP-MS experiments, 50% of the sample was loaded.

### Mass spectrometry data analysis

Peptides were identified and quantified using MaxQuant v 1.6.0.16 (Tyanova *et al.*, 2016a). The UniProt Arabidopsis protein database (downloaded on 01/2018, 41 350 entries) was used for spectrum-to-sequence matching. For the label free quantitative (LFQ) proteome data, search parameters included trypsin digest enzyme, allowing for up to two missed cleavages, Cys carbamidomethylation as fixed, and N-terminal acetylation and Met oxidation as variable modification. False discovery thresholds of 0.01 were applied at the level of peptide-spectrum matching (PSM) and for protein identifications, two quantification events were required for quantification, and the “match between runs” option in MaxQuant was enabled for replicates within each group/genotype. The “calculate iBAQ values” option was enabled. Further analysis was performed using the Perseus statistical software package v1.6.1.1 (Tyanova *et al.*, 2016b). Contaminants, reverse hits, and proteins identified only by modified peptides were excluded. Results were filtered for protein groups quantified in at least two out of four biological replicates in at least one genotype, LFQ intensities were log_2_-transformed, and missing values imputed using Perseus standard settings. Significant differences in protein accumulation were determined by ANOVA analysis with Benjamini-Hochberg false discover rate (FDR)<0.05, followed by Tukey´s honest significance test. Differentially regulated proteins were also clustered and analyzed for functional KEGG term enrichment and protein interactions using STRING db v.11, using a high confidence database, experimental and co-expression information only (Szklarczyk *et al.*, 2018). For N-terminome analysis, MaxQuant search parameters were adapted to semi-specific (free N-terminus) ArgC as digestion enzyme, isotope labeling by light (+28.031300) and/or heavy (+36.075670) dimethylation of Lys residues and peptide N termini, Cys carbamidomethylation as fixed and Met oxidation, N-terminal acetylation (+42.010565) or N-terminal pyroGlu formation from Glu (-18.010565) or Gln (-17.026549) as variable modifications (Hofsetz *et al.*, 2020). Identified N-termini were annotated based on the modification of SpecificPeptides.txt file of the MaxQuant output folder, with information from Uniprot.org, using an in-house script (MaxQuant Advanced N Termini Interpreter, MANTI.pl version 3.9.5, https://sourceforge.net/projects/manti/). The prediction of sub-cellular localization and signal peptide (SP) or transit peptide (TP) cleavage sites was retrieved from TargetP2.0 (Almagro Armenteros *et al.*, 2019). Protein termini were considered significantly changed in abundance between two genotypes if they passed a Linear Model for Microarray Analysis (LIMMA)-moderated *t*-test (Ritchie *et al.*, 2006; Gomez-Auli *et al.*, 2016) with *P*<0.05, and changed more than 50% in abundance (log_2_>0.58 or <–0.58).

## Results

### FtsH12 is expressed in non-photosynthetic plastids, located in the inner envelope with its C-terminus exposed to the stroma

While most active FtsH proteases in *Arabidopsis thaliana* arose after an evolutionary recent gene duplication event Wagner *et al.*, 2012, FtsH12 is not part of a homologous pair. This protease has a higher molecular weight (112 kDa) than the other active FtsH enzymes due to a longer N-terminal stretch (Supplementary Fig. S1A) containing two transmembrane helices (Supplementary Fig. S1B). Furthermore, three conserved amino acids in the Walker B motifs are exchanged (F587 for I) and second region of homology (SRH) (E651 for A and F652 for L), compared with the consensus sequence that might impair ATP hydrolysis (Supplementary Fig. S1C). In the catalytic HEXXH motif, described as “abXHEbbHbc”, the “a” in active metalloproteases is most often a valine or threonine, “b” an uncharged residue, and “c” a hydrophobic residue. In FtsH12, however, an unusual leucine is placed at the “a” site, according to Interproscan (Supplementary Fig. S1C). Arabidopsis eFP browser (data accessible at http://bar.utoronto.ca) indicates stronger expression of *FTSH12* and other subunits of the FtsH12/FtsHi complex in photosynthetic tissues, but also marked expression in roots, flowers and seeds (Supplementary Fig. S2A; for comparison, expression of the thylakoid-located FtsH*2* and of the import complex subunit *YCF1* are shown). GUS staining in *FTSH12* promoter fusion lines confirmed expression of *FTSH12* in young vegetative plant leaves and roots, as well as in generative organs and embryos (Supplementary Fig. S2B). Immunoblotting with a peptide-directed antibody raised against the C-terminal sequence DRVSYQPVDLRAAPLHRS of FtsH12 revealed steady abundance of this protease during leaf development, not only in chloroplasts, but also in non-photosynthetic organs, including roots (Supplementary Fig. S3A). In dark-grown *A. thaliana* cell cultures, where chloroplast development is delayed due to the lack of leaf tissue (Dubreuil *et al.*, 2018), FtsH12 was detected in proplastids, throughout all stages of plastid development and in mature chloroplasts (Supplementary Fig. S3B).

Based on limited cleavage by thermolysin in the protease protection assays with isolated pea chloroplasts, FtsH12 was reported to be located in the inner chloroplast envelope (Li *et al.*, 2017). However, thermolysin has been reported not to penetrate the outer chloroplast envelope (McAndrew *et al.*, 2001; Knopf *et al.*, 2012); limited cleavage would therefore indicate a localization in the outer chloroplast envelope. This ambiguity led us to reinvestigate biochemical fractions of chloroplasts. Compared with whole chloroplast extracts, the FtsH12 immunosignal strongly increased in envelope fractions, but was absent in samples of the thylakoid membrane (Supplementary Fig. S4A). The orientation of FtsH12 within the envelope membrane was further tested *in organello* protease protection assays. Treatment with thermolysin only slightly decreased the amount of mature FtsH12 (Supplementary Fig. S4B), most likely due to the presence of broken chloroplasts. In contrast, treatment of intact chloroplasts with trypsin, which is able to penetrate the outer envelope (McAndrew *et al.*, 2001), removed the protein band detected at 112 kDa. Both treatments lead to new digestion products with molecular masses of 60 kDa, consistent with a C-terminal fragment after truncation before the second transmembrane domain. Peptide competition demonstrated that these bands were specific for the FtsH12 epitope (Supplementary Fig. S4C). After solubilization of the membranes with Triton X-100, FtsH12 was completely degraded by both thermolysin and trypsin (Supplementary Fig. S4B). Together, this confirms that FtsH12 is located in the inner chloroplast envelope, with the C-terminal part containing the AAA-type domain and the catalytic domain facing the stroma, as previously suggested by Li *et al.* (2017) and confirmed by Schreier *et al.* (2018).

### FtsH12 forms a heteromeric complex with inactive FtsH subunits and plastid NAD-malate dehydrogenase

Homozygous *ftsh12* knock-out lines arrest embryo development at the heart stage (Meinke *et al.*, 2008). To determine native interaction partners of FtsH12, we generated a stable insertion line expressing FtsH12 MYC-tagged at its C-terminus under control of the native FtsH12 promoter (P12::*ftsH*12CDS::4×c-myc; Supplementary Fig. S5) in the background of a mutant line carrying a T-DNA insertion in the seventh exon of *FTSH12*. Expression of MYC-tagged FtsH12 allowed segregation of a viable homozygous *ftsh12* T-DNA insertion line with no visible phenotype, indicating full complementation and a normal function of MYC-tagged FtsH12. FtsH12 complexes were isolated from chloroplasts of homozygous P12::*ftsH*12CDS::4×c-myc plants lacking endogenous FtsH12, by immunoprecipitation with anti-MYC antibody attached to magnetic beads and analyzed by mass spectrometry. As negative controls, pull-down experiments were performed with a MYC-tag using wild type chloroplasts, and pull-down by His-tag using the p*FTSH12*:*FTSH*12::6×Myc lines (Supplementary Fig. S6). Proteins with annotated chloroplast location identified in both biological replicates, but not the control samples, were considered as strong candidate interaction partners, and are shown in Table 1. FtsH12 was identified with most unique peptides and strongest intensity, followed by plastid malate dehydrogenase, FtsHi1, and FtsHi5. Additionally, two ribosomal proteins co-immunoprecipitated strongly with FtsH12 (Table 1). Also, FtsHi2 and FtsHi4 co-immunoprecipitated with FtsH12, but failed to meet our criteria as they were only detected in one of the two replicate experiments with P12::*ftsH*12CDS::4×c-myc (Supplementary Table S2). Nevertheless, this indicates that all four FtsHi homologs and pdNAD-MDH form a complex with FtsH12 (Schreier *et al.*, 2018), even when FtsH12 is expressed under its native promotor, whereas we did not detect YCF2 after immunoprecipitation of FtsH12 expressed under its native promoter and the chosen isolation conditions.

**Table 1.** **Stringent subset after immuno co-precipitation of myc-tagged FtsH12 expressed under its native promoter.** Two independent experiments were performed on two FtsH12-4x-cMYC expressing lines, proteins were identified and quantified by LC-MS/MS; only putative interactors observed in both replicate pull down experiments are shown. UniProt-annotated names and accession numbers, number of unique peptide sequences found for each protein group, sequence coverage in percent, and log_2_ transformed MaxQuant LFQ intensity for each experiment are listed.

Names	UniProt ID	Unique peptides	Sequence coverage [%]	log_2_(LFQ) FtsH12-4xcmyc replicate 1	log_2_(LFQ) FtsH12- 4xcmyc replicate 2
FtsH12-4xcMYC		12	13.7	20.7	19.1
Malate dehydrogenase, chloroplastic	Q9SN86	7	23.8	18.9	20.5
50S ribosomal protein L12,chloroplastic	P36212; P36210	3	36.4	18.5	20.5
30S ribosomal protein S19, chloroplastic	P56808	2	20.7	17.0	19.2
Inactive ATP-dependent zinc metalloprotease FtsHi5	F4J3N2	4	5.1	16.3	19.5
Inactive ATP-dependent zinc metalloprotease FtsHi1	O22993	7	10.3	19.0	16.4

**Fig. 1.**
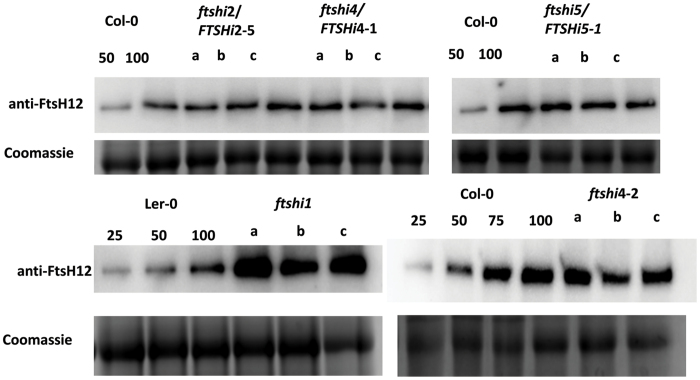
Expression of *FTSH12* in T-DNA inserted *FTSHi* mutant lines. Immunostaining of FtsH12 in total protein extracts of 10 day-old *FTSHi* mutant seedlings grown on MS-media growth plates. FtsH12 protein amount is displayed in the heterozygous mutants *ftshi2-5, 4-1* and *5-1* (upper panel) and in the homozygous mutants *ftshi1-1* and *ftshi4-2* (lower panel). Information on the *FTSHi* mutant lines can be found in (Mishra *et al.*, 2019). A total of 15 µg protein was loaded from the *FTSHi* mutant lines and wild type (100%); for comparison wild type was additionally loaded at lower concentrations (25, 50, and 75%). a, b, c correspond to three biological replicates.

To analyse if the presence of the inactive FtsHi enzymes had impact on the FtsH12/FtsHi1,2,4,5 complex accumulation, FtsH12 was immuno-stained in the heterozygous *FTSHi* mutants *ftshi2*/FTSHi2-5, *ftshi4*/FTSHi4-1 and *ftshi5*/FTSHi5-1 (Mishra *et al.*, 2019), as well as in the homozygous mutants *ftshi1* and *ftshi4-2* (Mishra *et al.*, 2019), and their respective wild type controls (Fig. 1; Supplementary Table S3). While the amount of FtsH12 seemed to be similar in wild type and the heterozygous mutants, in *ftshi1* the amount of FtsH12 was strongly up-regulated. In *ftshi4-2*, however, the amount of FtsH12 was slightly diminished.

### Reduced amount of FtsH12 cause changes in leaf phenotype, pigmentation and plastid ultrastructure

**Fig. 2.**
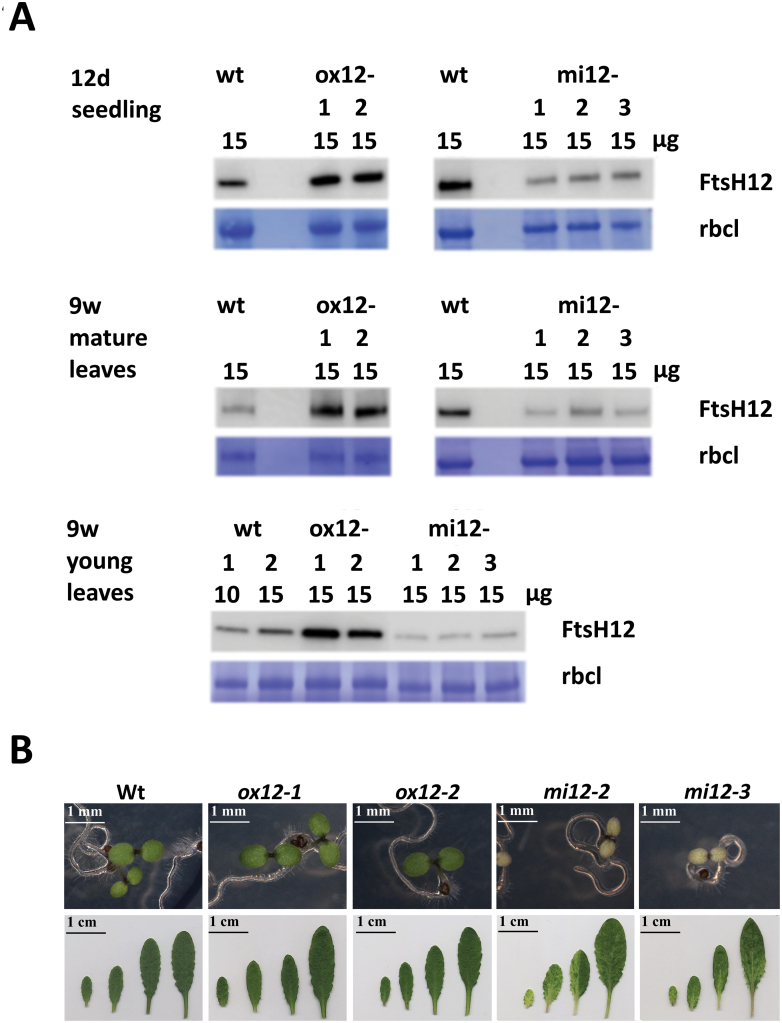
Phenotypic comparison of wild type (WT), *FTSH12* overexpressor and knock-down plants. (A) Immunostain to quantify FtsH12 protein amounts in leaves of wild type and independent transgenic lines at 12 days (12 d) and nine weeks (9 w) of age grown in short light regime (8 h of light per day). Rosette leaves of nine-week-old plants were analysed separately for developing (young) and mature leaves. Amounts of total proteins loaded on SDS PAGE are given above each lane. At least three biological replicates were tested. As loading control the Coomassie-stained band of large subunit of RuBisCo (rbcL) is shown. (B) Phenotypes of *A. thaliana* wild type and transgenic lines with increased (*ox12-1* and *ox12-2*), or reduced (*mi12-2* and *mi12-3*) *FTSH12* expression grown in short day light regime (8 h of light per day). Two-day-old seedlings are shown in the upper panel (scale bar represents 1 mm), and leaves of nine-week-old plants in the lower panel (scale bar represents 1 cm).

To gain insights into the physiological functions of FtsH12, we generated micro-RNA lines with reduced amounts of FtsH12, as well as *FTSH12* overexpressor lines (*S35::ftsH12CDS)* in *A. thaliana* Col-0 background. Two independent homozygous *FTSH12* overexpressor lines (*ox12-1*, *ox12-2*) and three independent micro-RNA lines (indicated as *mi12-1*, *mi12-2* and *mi12-3* in the protein domain structure of Supplementary Fig. S1A) were selected for further experiments. Immunoblot analyses using the FtsH12 antibody showed a reduction of FtsH12 in all *mi12* lines by 50–60% compared with that seen in wild type, while *ox12* lines showed increased amounts of FtsH12 by 200–400% compared with wild type grown in short day conditions (Fig. 2A), independent of the age of plants or leaves. Strong phenotypic differences were observed in the *FTSH12* knock-down plants: Two days after germination in light, the *mi12* lines displayed pale yellow cotyledons (Fig. 2B, upper panel), in which chlorophyll accumulation was retained. In nine-week-old plants of *mi12* lines, a variegation pattern of light yellow-green and dark green areas was observed in young developing leaves, while mature leaves contained almost no variegated areas (Fig. 2B, lower panel). Consistently, significantly reduced Chl *a* and Chl *b* content was found in young *mi12* leaves (Supplementary Fig. S7C). Mature *mi12* plants appeared slightly smaller, but this difference in their rosette size was not significant (Supplementary Fig. S7A). Furthermore, their cotyledon tip-to-tip distance was significantly reduced compared with the wild type (Supplementary Fig. S7B). Maximal PSII quantum yields and non-photochemical quenching in fully developed leaves of *mi12* lines were indistinguishable from wild type (Supplementary Table S4). In contrast to *FTSH12* knock-down, *ox12* lines with increased amounts of FtsH12 showed larger cotyledon tip-to-tip distance and higher chlorophyll content in mature leaves, but none of these apparent differences were significant compared with wild type (Supplementary Fig. S7A, B and Table S4). No obvious changes in superoxide anion or hydrogen peroxide concentrations were observed in 10-day-old wild type or mutant seedlings grown either in short day or in continuous light (Supplementary Fig. S8).

**Fig. 3.**
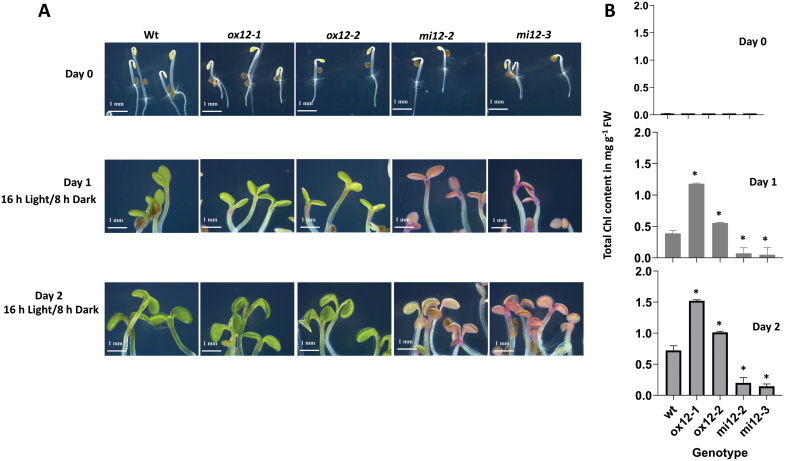
De-etiolation of wild type (WT), *FTSH12* overexpressor and knock-down plants. (A) Greening of wild type and transgenic lines (*ox12-1*, *ox12-2* and *mi12-2*, *mi12-3*) after etiolated growth (0 h light, top panel) for five to six days followed by illumination (16 h light/ 8 h dark) for 1 d (middle panel) and 2 d (bottom panel). Scale bar=1 mm. (B) Quantification of chlorophyll content in wild type and mutant lines during de-etiolation. Significant differences between wild type and transgenic plants are indicated by asterisk (Student’s t-test, *P* < 0.05).

To analyse if *FTSH12* overexpression had the potential to accelerate chloroplast development, wild type and *FTSH12* mutant lines were exposed to de-etiolation; under these more challenging conditions *FTSH12* overexpression might provide an advantage (Fig. 3A; Supplementary Fig. S7D, E). The *FTSH12* knock-down lines *mi12-1* and *mi12-2* remained smaller (Supplementary Fig. S7E), accumulated anthocyanins (Supplementary Fig. S7D), and therefore had a pink appearance (Fig. 3A). Interestingly, the overexpressor lines contained significantly more chlorophyll during day 1 and day 2 of de-etiolation than wild type (Fig. 3B, right panel).

**Fig. 4.**
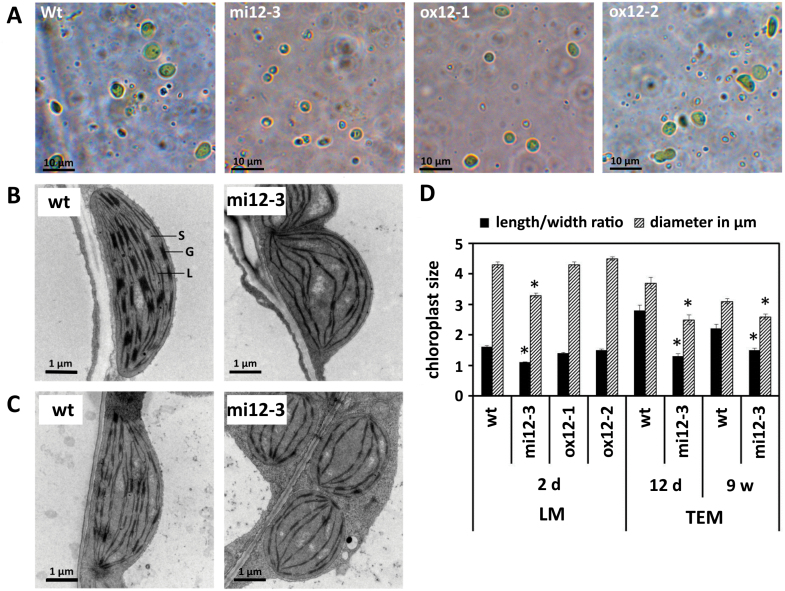
Size and morphology of chloroplasts. (A) Light microscope (LM) micrographs of isolated chloroplasts of two-day-old wild type (WT) seedlings and *FTSH12* mutant lines in isosmotic buffer visualized with a Zeiss Axioplan 2 at 1000 × magnification. (B, C) Transmission electron micrographs (TEM) showing chloroplasts of wild type and of the *FTSH12* knock-down line (*mi12-3*, variegated leaf areas) at the age of 12 d (B) and nine weeks (C). Visible are the chloroplast stroma (S) containing the thylakoid membrane consisting of dense photosystem II-containing grana stacks (G) and photosystem I-enriched stroma lamellae (L). (D) Average diameters (grey bars, ±SE) and length to width ratios (black bars, ±SE) of chloroplasts are shown for each genotype. Significant differences compared with wild type are indicated by an asterisk (n>35 for light microscope pictures, n>15 for TEM, *P*<0.05, Student´s *t*-test).

Chloroplasts of two-day-old seedlings of wild type, overexpressor (*ox12-1*, *ox12-2*) and knock-down (*mi12-3*) lines were isolated and their sizes analysed in isosmotic medium by light microscopy (Fig. 4A). In addition the morphology of chloroplasts in variegated leaf areas of young (12 d) and old (nine weeks) *mi12* plants (Fig. 4B, C) were analysed by transmission electron microscopy (TEM). While wild type and the overexpressor lines displayed length/width (l/w) ratios of 1.4 and 1.5, chloroplasts isolated from *mi12* were more spherical with a significantly lower l/w ratio of 1.1. Also the average diameter of chloroplasts in the *FTSH12* knock-down mutant was significantly reduced to 3.3 µm, compared with 4.3 µm in wild type and *ox12* lines (Fig. 4D). In the TEM analysis, wild type chloroplasts displayed a l/w ratio of 2.2, while chloroplasts of the *mi12-3* line had a l/w ratio of 1.5, confirming the results obtained by light microscopy (LM) of isolated chloroplasts (Fig. 4D). Furthermore, a smaller fraction of the thylakoid membranes in the *FTSH12* knock-down plants appeared stacked into grana lamellae, with fewer and lower stacks compared with wild type. Irrespective of the plant age, the distances between the thylakoid membranes (stroma lamellae and grana stacks) seemed enlarged in *mi12-3* (Fig. 4B, C).

### Altered FtsH12 amounts affect translocon at the inner chloroplast envelope membrane composition

To obtain a global view on differences in protein abundance, proteomes extracted from four biological replicates of two-day-old wild type, *ox12-1* and *mi12-3* seedlings were analysed. Label-free quantitative mass spectrometry was used to identify 4061 proteins, of which 3129 could be quantified in at least two replicate experiments of one genotype (Supplementary Table S5). This included FtsH12, which showed modest abundance in wild type, massive accumulation in the overexpressor line, and abundance below detection limit in the miRNA line, as judged by intensity-based absolute quantification (iBAQ) values (Table 2). Note that the relative FtsH12 amounts suffer from a bias, as only a single FtsH12 peptide was quantified in wild type, but 14 were quantified in the overexpressor line, resulting in higher summed intensity and exaggerated iBAQ. Given the proposed function of FtsH12 as part of an import motor protein complex, we further analysed the behavior of the FtsH12-complex subunits, TIC components and other import-associated proteins identified in our dataset, as summarized in Table 2. The FtsHi subunits reflected FtsH12 amounts and were not detected in *mi12-3*, but significantly accumulated in *ox12-1*. YCF2 was significantly reduced in *mi12-3*, but not up-regulated in *ox12-1*, while the amount of the abundant plastidial NAD-MDH did not differ in *mi12-3* and wild type. Subunits of the 1 MDa TIC complex comprising Tic100, Tic56, YCF1/Tic214 and Tic20-I (Nakai, 2018), that has been shown to interact with the FtsH12 complex (Kikuchi *et al.*, 2018), appeared to accumulate in *mi12-3* compared with wild type. Notably, a complementing trend was reported for depletion of Tic56, which increased the abundance of the FtsH12 complex (Schäfer *et al.*, 2019). Amongst the other identified TIC subunits, only TIC22-III showed significant accumulation in *mi12-3* compared with wild type, whilst all other identified TIC subunits and chaperones considered as plastid import motor components accumulated only in *ox12-1* compared with wild type (Table 2). To investigate the cause of these striking changes in TIC complex subunit abundance, we next analysed RNA extracted from two day- and six-week-old Arabidopsis leaves by qPCR (Supplementary Fig. S9). In 2 d-old seedlings, the transcripts of *YCF1*, *TIC100,* and *TIC56,* as well as *TIC110* and *TIC40,* were strongly up-regulated in the overexpressor line *ox12-1* compared with wild type, but also, to a lesser extent, in the knock-down line *mi12-3*. In leaves of six-week-old plants, however, only *mi12-3* expressed *TIC100*, *TIC110* and *TIC40* stronger than wild type, while expression of all complex subunits was reduced in *ox12-1* compared with wild type. Taken together, these results suggests that FtsH12 abundance determines the accumulation of the FtsH12-FtsHi-pNAD-MDH complex, and further affects the abundance of the 1 MDa TIC complex by transcriptional changes.

**Table 2.** **Comparison of FtsH12 and TIC complex protein abundance in *FTSH12* mutants.** Protein abundance in *FtsH12* knock-down plants (*mi12-3*), overexpressors (*ox12-1*) and wild type (WT) was determined by label-free mass spectrometry. Listed are protein names with gene loci and associated Uniprot IDs, number of unique peptides supporting the identification, and average iBAQ values as a measure of protein abundance. Significance between FTSH mutants and wild type was determined by a two-tailed Student’s *t*-test (*P*<0.05)

Protein name	Gene loci	UniProt IDs	Unique peptides	Average iBAQ *mi12-3*	Average iBAQ WT	Average iBAQ *ox12-1*	Significant WT/mi12-3	Significant *ox12-1*/*mi12-3*	Significant *ox12-1*/WT
***FtsH12 motor complex subunits***									
FTSH12	At1g79560	Q9SAJ3	14	0	3110	87 893	+	+	+
FtsHi1	At4g23940	O22993	6	0	2134	8014	+	+	+
FtsHi2	At3g16290	A8MPR5	4	0	3824	8437	+	+	+
FtsHi4	At5g64580	F4KF14	3	0	3992	6308	+	+	+
FtsHi5	At3g04340	F4J3N2	2	0	659	2509		+	+
YCF2	AtCg00860;AtCg01280	P56786	5	1433	2470	1453	+		
pdNAD-MDH	At3g47520	Q9SN86	13	1 270 450	1 093 783	1 718 300		+	+
***1MD TIC complex***									
TIC214 / YCF1	AtCg01000;AtCg01130	P56785	14	20 821	5097	19 452	+		+
TIC100	At5g22640	Q8LPR8	7	16 646	3752	33 105	+		+
TIC56	At5g01590	Q7Y1W1	6	19 101	7802	46 503			+
***Other TIC subunits***									
TIC110	At1g06950	Q8LPR9	43	568 540	396 450	667 520			+
TIC40	At5g16620	Q9FMD5	6	105 910	83 516	123 841			+
TIC62	At3g18890	Q8H0U5	6	0	0	23 804		+	+
TIC55	At2g24820	Q9SK50	8	10 723	8683	40 684		+	+
TIC22-IV	At4g33350	Q9SZB2	5	29 746	24 446	49 074		+	+
TIC22-III	At3g23710	F4J469	2	20 041	5375	10 095	+	+	
***Chloroplast chaperones with suggested motor function***									
cpHsc70-1	At4g24280	Q9STW6	9	1 728 700	1 531 400	2 821 950		+	+
cpHsc70-2	At5g49910	Q9LTX9	9	188 855	207 958	395 635		+	+
HSP93-III/ CLPC2	At3g48870	F4JF64;Q9SXJ7	12	176 430	168 343	250 565		+	+
CLPC1	At5g50920	Q9FI56	13	1 257 550	1 220 073	2 193 150		+	+

### Seedlings with modulated FtsH12 content show drastic changes in proteome composition

**Fig. 5.**
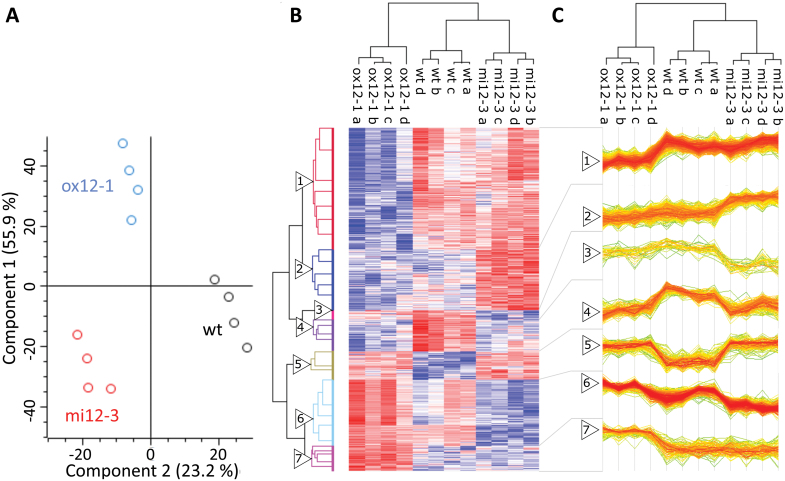
Label free quantitative proteome analysis of two-day-old wild type (WT), *ox12-1* and *mi12-3* seedlings. (A) Principal component analysis (PCA) of 1450 proteins with significant differences in abundance between the three lines. Four biological replicates were analysed for each line, differences determined by multi-sample ANOVA with Benjamini-Hochberg correction for multiple hypothesis testing (FDR<0.05). WT: Black circles, *mi12*: red circles, *ox12*: blue circles. (B) Hierarchical clustering of the 1450 proteins with significant differences in abundance after Z-score normalization. Blue color of the heat map indicates LFQ intensities below, and red color, above average for each protein. (C) Expression patterns of the seven clusters with similar protein abundance profiles determined by hierarchical clustering in (B), with more intense red indicating larger number of overlaying expression profiles.

The quantitative proteome analysis further indicated massive changes in proteome composition in *FTSH12* transgenic plants, with 1450 of the 3129 quantified proteins exhibiting significant differences in abundance (ANOVA with Benjamini-Hochberg FDR<0.05, followed by Tukey´s honest significance test; Benjamini and Hochberg, 1995; Supplementary Tables S5 and S6). Principal component analysis (PCA) clearly separated the data into three distinct clusters representing wild type, *mi12-3*, and *ox12-1*, verifying that the observed changes were indeed linked to the genotypes (Fig. 5A). Hierarchical clustering of z-score normalized intensities of proteins with differential abundance revealed seven clusters with similar protein accumulation profiles (Fig. 5B, C, Supplementary Table S5); these were further analysed for enriched KEGG functional categories and known interactions among the cluster constituents, using the String database (Szklarczyk *et al.*, 2018). The large cluster 1 contained 514 highly connected proteins with lower abundance in *ox12-1* compared with wild type and miRNA knock-down (Fig. 5C). KEGG term enrichment indicated that this cluster included many of the proteins involved in central carbon metabolism or protein synthesis and quality control, such as the spliceosome, ribosome, proteasome and reticulum proteins (Supplementary Fig. S10). Cluster 2 consisted of 256 proteins with higher accumulation in *mi12-3*, but no significant difference between *ox12-1* and wild type, which included additional proteasomal subunits, proteins involved in carbon metabolism, and phagocytosis (Supplementary Fig. S11). Also, proteins involved in fatty acid metabolism, such as acetyl coenzyme A carboxylase 1 (ACC1) and fatty acid export 1 (FAX1), and the translocon on the outer chloroplast membrane (TOC) 34, translocon on the inner chloroplast membrane (TIC) 22L and 110 and the chloroplastic import inner membrane translocase subunit HP30-2, components of the plastid protein import machinery, were found in this cluster (Supplementary Table S5). Notably, clusters 1 and 2 together contained 75 proteins that are commonly observed in albino/green pale mutants, indicating general proteome changes associated with impaired photosynthesis (Motohashi *et al.*, 2012). A small cluster 3 contained 38 proteins with reduced abundance in *mi12-3,* compared with the other two genotypes, including YCF2, but it was too heterogeneous to reveal enriched KEGG terms. 

GO term analysis showed the presence of a small cluster of four RNA helicase-associated proteins (Supplementary Fig. S12). Cluster 4 consisted of 135 proteins that were less abundant in both *FTSH12* mutants, *oxp12-1* and *mi12-1*, compared with wild type. This cluster included 20 subunits of the cytosolic ribosome complex (Supplementary Fig. S13). Cluster 5 contained 121 proteins with higher abundance in both *FTSH12* mutants, including an additional nine ribosomal proteins (Supplementary Fig. S14), but also the plastid RNA polymerase subunits RPOA and RPOB. Cluster 6 contained 279 proteins with highest abundance observed in *ox12-1,* and lowest abundance in *mi12-3,* and therefore correlated with the amount of FtsH12. This cluster was formed by chloroplast proteins highly connected with each other in the graphical depiction (Supplementary Fig. S15), and with KEGG-annotated functions in photosynthesis, photosynthetic carbon fixation, chlorophyll biosynthesis, and plastid protein synthesis. Finally, cluster 7 contained 107 proteins with distinctly higher abundance in *ox12-1* compared with wild type and *mi12-3*, including many thylakoid proteins, the plastid envelope and stromal proteins TIC62, TIC55, YCF3, and chlorophyll synthase (Supplementary Table S6), and also subunits of the plastid ribosome (Supplementary Fig. S16). Taken together, clusters 6 and 7 reflected the accelerated development of photosynthetic capacity in *ox12-1* and wild type, compared with *mi12-3* seedlings observed at this stage.

### N-terminome analysis shows normal maturation of plastid-imported proteins in *FtsH12* mutants

**Fig. 6.**
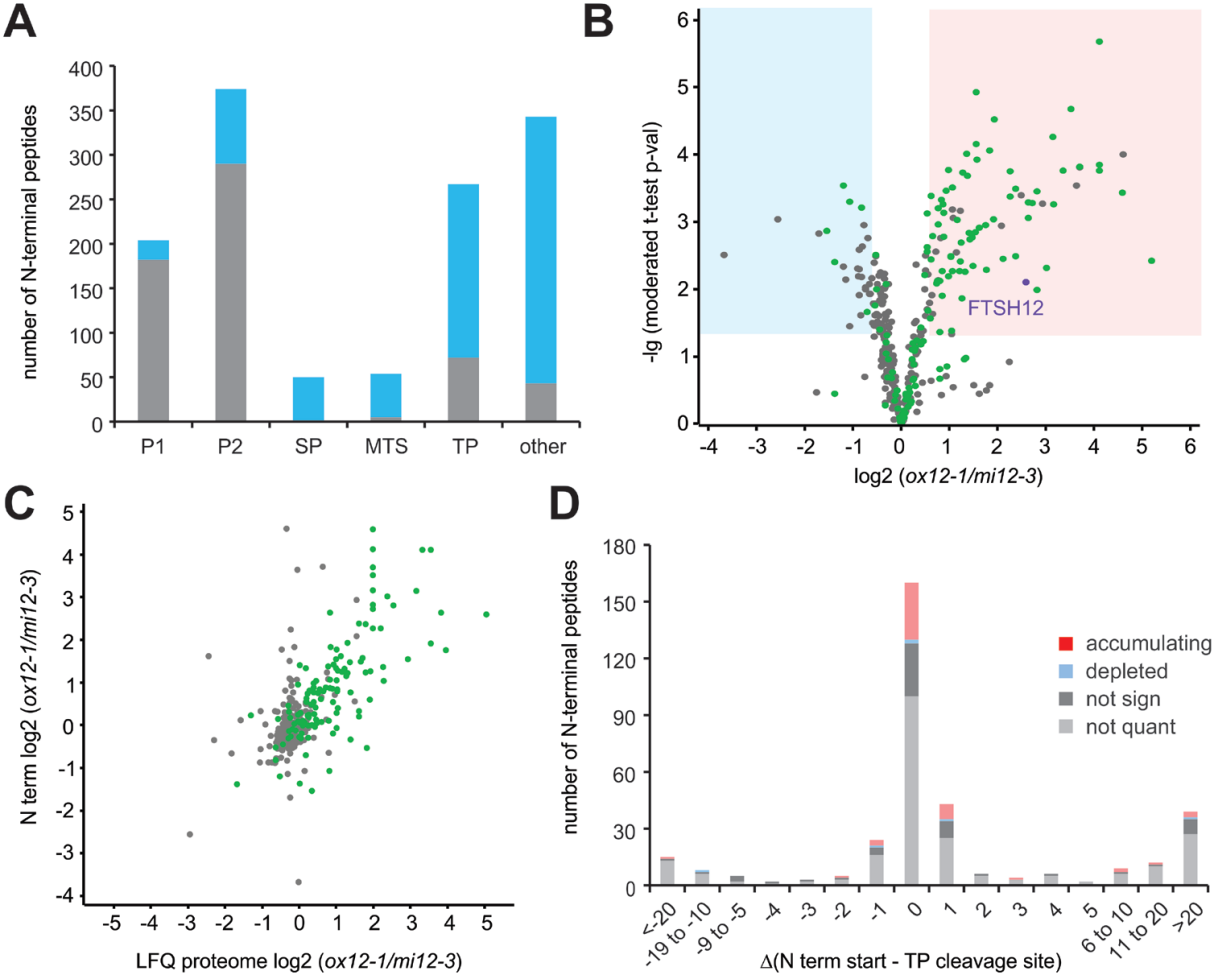
Quantitative N-terminome analysis in *FTSH12* mutants compared with wild type. (A) Positional annotation of 1292 identified N-terminal peptides. Peptides mapping to translation start sites with intact or excised initiator-Met (Pos1/2) or within five amino acids of TargetP2.0 annotated as signal peptide, plastid or mitochondrial transit peptide or propeptide maturation sites, were defined as ‘expected’ N termini. Light grey, peptides with endogenous N-terminal acetylation; dark grey, peptides *in vitro* marked by dimethylation, indicating *in vivo* free N-terminal amines. (B) Volcano plots of N-terminal peptide abundance in *ox12-1* compared with *mi12-3*. Peptides from plastid-located proteins (plastid encoded or predicted to target to the plastid by TargetP2.0) are highlighted in green. N-terminal peptides with significantly reduced or increased abundance (Student´s *t*-test *P*-value<0.05 and log_2_ FC<–0.58 or >0.58) are highlighted with blue and red color background. (C) Comparison of N-terminal peptide abundance with the corresponding protein abundance within *ox12-1* and *mi12-3.* Peptides from plastid proteins are highlighted in green. Pearson correlation coefficient with confidence level is indicated. (D) Comparison of the position of plastid protein N-terminal peptides to the transit peptide cleavage predicted by TargetP2.0. Protein termini showing significant accumulation or depletion change in *ox12-1* compared with *mi12-3* are highlighted in red and blue, respectively. Dark grey indicates unaltered abundance, light grey indicates peptides not quantified in two or more replicates.

Given the reduced abundance of nuclear-encoded, plastid-imported proteins, we considered whether altered FtsH12 abundance would result in differential processing of nuclear-encoded proteins after plastid import. To address this problem, we profiled N termini of proteins in the same samples using undecanal-assisted N-termini enrichment, in an early version of our recently published HUNTER protocol (Weng *et al.*, 2019). Free primary amines, including α-amines of protein N-termini generated by *in vivo* proteolytic processing, were modified by reductive dimethylation using distinct formaldehyde isotopes for each line. Tryptic digest then generated new primary amines on non-N-terminal peptides that were subsequently tagged with long-chain hydrophobic aldehyde, allowing their removal from both endogenously modified and *in vitro* dimethylated N-terminal peptides by reverse-phase C18 solid phase extraction. Mass spectrometric analysis identified 1292 N-terminal peptides from 498 proteins, including 592 N-terminal peptides modified by endogenous N-terminal acetylation and 700 dimethylated peptides representing N termini with free α-amines *in vivo* (Supplementary Table S7). Positional annotation revealed that the majority of the N-terminal peptides matched the protein start site with intact or excised initiator Met, positions within five amino acids distance from sites of signal peptide cleavage in the secretory pathway, or transit peptide cleavage after import into mitochondria or chloroplasts, as predicted by TargetP2.0 (Almagro Armenteros *et al.*, 2019; Fig. 6A). Of all identified N-terminal peptides, 343 (27%), including 91 peptides from proteins with predicted plastid TP (Transit peptide), matched to sites within the protein models that have not been previously annotated (Fig. 6A). As expected, the majority of the N-terminal peptides at positions 1 (89%) and 2 (78%) were N-terminally acetylated, as were a large proportion (27%) of the mature plastid N-termini. However, only few mitochondrial protein N-termini, and none of the signal peptide-processed N-termini were N-terminally acetylated. Out of the unannotated N-terminal peptides, 43 were acetylated, which indicates proteoforms arising from alternative splicing, use of alternative translation initiation sites, or rare post-translational N-terminal acetylation after proteolytic processing (Perrar *et al.*, 2019).

To determine significantly altered protein termini abundance that may indicate differential cleavage between two genotypes, we filtered the dataset for N-terminal peptides changing more than 50% in abundance (log_2_ (FC)>0.58 or <–0.58) that were supported by a LIMMA-moderated *t*-test (*P*-value<0.05; Gomez-Auli *et al.*, 2016; Hartl *et al.*, 2017). Application of these criteria to compare plants with highest and lowest abundance of FtsH12 identified 86 N-terminal peptides with significantly higher abundance in *ox12-1* than in *mi12-3* (Fig. 6B; Supplementary Table S7), of which 69 belonged to 62 plastid proteins. Expected positions at the translation start site of plastid-encoded proteins or predicted TP cleavage sites were matched for 53 significantly changing N-terminal peptides, which included the mature dimethylated N-terminus of FtsH12 starting at Ser51 after TP cleavage (Fig. 6B). In contrast, 29 N-terminal peptides, including only six from plastid-imported proteins, were more abundant in the knock-down *mi12-3* compared with the overexpressor line *ox12-1* (Fig. 6C). Comparison of wild type with *mi12-3* (Supplementary Fig. S17A), and *ox12-1* with wild type revealed similar results (Supplementary Fig. S17B). Thus, both expected and unexpected plastid protein N-termini were consistently more abundant in the plant line with higher FtsH12 content in each comparison. Consistently, we observed a significant correlation between N-termini abundance and the corresponding total protein abundance determined by label-free quantitation in all three comparisons of *ox12-1* and *mi12-3* (Pearson correlation r=0.67; Fig. 6C), *mi12-3* and wild type (r=0.61; Supplementary Fig. S17C) and *ox12-1* and wild type (r=0.70; Supplementary Fig. S17D). This indicated that the majority of the changes in N-termini abundance reflected the changes in overall proteome composition. A few N-terminal peptides showed deviating behavior, with significant changes in *ox12-1* or wild type compared with *mi12-3,* and unchanged protein abundance. However, on manual inspection these were found either not to be plastid-located and/or arising from low abundance proteins, where imputation compressed or masked the true protein ratio in the proteome dataset. Therefore, no strong candidates for direct FtsH12-mediated proteolytic cleavage were observed. We further speculated that altered FtsH12 activity might alter protein maturation after import. Most of the identified N-termini of nuclear-encoded plastid-targeted proteins matched chloroplast TP cleavage sites predicted by TargetP2.0, or were shifted by one or two residues towards either site (Fig. 6D), while N-terminal peptides resulted from proteolytic cleavage within the protein. The latter may reflect functional processing, for example, the eight identified lumen TP cleavages (Supplementary Table S7), or represent degradation intermediates of abundant proteins. Similar “ragged” plastid protein N-termini and proteolytic fragments have previously been observed by detailed plastid N-terminome profiling (Rowland *et al.*, 2015). Only 25 N-terminal peptides of plastid proteins matched to positions more than five amino acids preceding the predicted transit-peptide cleavage sites, including 12 N-termini mapping to position 2 of the protein model, indicating intact full-length precursor proteins (Supplementary Table S7). All of these were N-terminally acetylated at P2 after Met removal, but only the N-terminal peptide of an uncharacterized precursor protein encoded by gene At4g28440 contained a labeled Lys residue that allowed quantification in our assay, but was not significantly altered between the mutants.

## Discussion

Seed development of homozygous *ftsh12* knock-out plants is known to arrest at the heart stage of development (Meinke *et al.*, 2008). Consistent with an important general function in plastids and plastid development, we have shown that *FTSH12* is expressed throughout chloroplast development in synchronized cell cultures (Supplementary Fig. S3B), and also in non-photosynthetic proplastids and roots (Supplementary Fig. S3A). Biochemical fractionation of isolated chloroplasts further demonstrated that FtsH12 resides in the inner chloroplast envelope (Supplementary Fig. S4). In a previous study, FtsH12 was synthesized *in vitro* and imported into isolated pea chloroplasts (Li *et al.*, 2017). Subsequent treatment with thermolysin, a protease reported to be unable to penetrate the outer chloroplast envelope membrane (Knopf *et al.*, 2012), yielded a 39 kDa FtsH12 fragment, which would suggest that both N- and C-termini of FtsH12 protrude into the cytosol. In our assays, an antibody directed against the C-terminus of FtsH12 immuno-stained a band of 60 kDa after treatment with trypsin (Supplementary Fig. S4), a protease that penetrates the outer envelope membranes (McAndrew *et al.*, 2001). To some extent, the same fragment was observed after thermolysin treatment, presumably due to the presence of plastids with disrupted outer envelopes. FtsH12 was completely degraded by both proteases when detergent was added, suggesting that its catalytic and ATPase domain face the stroma, in agreement with more recent similar data (Kikuchi *et al.*, 2018; Schreier *et al.*, 2018). Together, this unambiguously confirms the localization of FtsH12 in the inner chloroplast envelope, as suggested previously (Li *et al.*, 2017). The observed sensitivity of FtsH12 to thermolysin treatment after *in vitro* import into pea chloroplasts (Li *et al.*, 2017), contrary to the expected behavior of inner envelope proteins in these assays (McAndrew *et al.*, 2001; Knopf *et al.*, 2012), may be due to (i) incorrect integration of pre-FtsH12, (ii) partially disrupted outer envelope, or (iii) a different resistance to thermolysin penetration in pea compared with Arabidopsis chloroplasts.

Complementation of an embryo-lethal *FTSH12* T-DNA insertion line allowed us to segregate a mutant line solely by expressing Myc-tagged FtsH12 under its native promoter. We used this line to study the native FtsH12 interaction partners without the presence of potentially interfering wild type protein, or side effects arising from overexpression. In two independent experiments, we were able to pull-down FtsH12 together with FtsHi1, FtsHi5, and the plastidic form of MDH from isolated chloroplasts, with FtsHi2 and FtsHi4 additionally found in one experiment (Supplementary Table S2). This is in agreement with data obtained by others, where a heteromeric complex comprised of FtsH12, FtsHi1, FtsHi2, FtsHi4, FtsHi5, pdNAD-MDH, and YCF2 was identified in co-immunoprecipitation experiments using Arabidopsis plants expressing FtsH12 or pdNAD-MDG as YFP fusion proteins under the control of the 35S promotor (Schreier *et al.*, 2018), and from tobacco chloroplasts expressing YCF2 as a HA-fusion protein (Kikuchi *et al.*, 2018). This large heteromeric 2 MDa complex was further shown to associate with a TIC complex comprised of TIC20, TIC56, TIC100 and TIC214/YCF1, driving protein translocation by the pulling force generated by the ATPase domains of the FtsHi subunits (Kikuchi *et al.*, 2018; Schreier *et al.*, 2018). However, these studies did not determine the stoichiometry or subunit arrangement of the FtsH12 motor complex. In our hands, expression of FtsH12 was up-regulated in single knock-down *FTSHi* mutants at the transcript (Mishra *et al.*, 2019) and protein (Fig. 1) level; in particular, the expression of FtsHi1 and FtsHi5 were dependent on the presence/absence of FtsH12. Both, FtsHi1 as well as FtsHi5 appeared most tightly bound to FtsH12 in our pull-down experiments (Supplementary Table S2). Given the strong expression of FtsH12 in the homozygous *ftshi1* mutant, it is tempting to speculate that FtsH12 may be able to substitute for FtsHi1 in the protein complex.

To assess the effect of modulated FtsH12 abundance, we created transgenic miRNA knock-down lines with reduced *FTSH12* expression (<40% of wild type), and overexpressor lines with increased *FTSH12* content (>3× that in wild type; Fig. 2A). The most obvious and striking phenotype in our *FTSH12* knock-down mutants were marbled (variegated) primary and young rosette leaves, dramatically altered chloroplast ultrastructure with deformed thylakoid membranes, and lower overall chlorophyll content. Similar pale-green or variegated phenotypes with altered chloroplast ultrastructure have also been observed in knock-down lines of *FTSHi4*, *FTSHi5* (Lu *et al.*, 2014; Wang *et al.*, 2018) and *pdNAD-MDH* (Schreier *et al.*, 2018), and in a line carrying a point mutation in *ftshi1* that renders its ATPase domain inactive (*arc1*; Kadirjan-Kalbach *et al.*, 2012; Mishra *et al.*, 2019), consistent with an important function of the FtsH12 complex in chloroplast development (Kikuchi *et al.*, 2018; Schreier *et al.*, 2018). The *arc1* mutant of FtsHi1 further contained smaller, more numerous chloroplasts, while overexpression of *FTSHi1* resulted in fewer, but larger chloroplasts (Kadirjan-Kalbach *et al.*, 2012). Similarly, a significant reduction in chloroplast size was observed in our *FTSH12* knock-down plants (Fig. 4), but our *FTSH12* overexpressor lines did not display significantly larger chloroplasts than wild type. Interestingly, when investigated under challenging conditions like de-etiolation (Fig. 3C), the overexpressor contained significantly more chlorophyll after one or two days in light, but did not grow faster. Even though phenotypic differences between *FTSH12* knock-down, wild type and overexpressor lines were most notable in early stages of plant development, we observed a delay of plastid development also during vegetative growth in developing leaves of *FTSH12* knock-down lines, suggesting that the FtsH12 complex is important at all developmental stages. This is consistent with the observation of pale-green and variegated leaves after depletion of *FTSHi5* by inducible expression of *FTSHi5*-targeting miRNA (Wang *et al.*, 2018). However, these authors observed increased amounts of H_2_O_2_ in *ftshi5-1*, while in *FTSH12* knock-down and overexpressor lines the occurrence of ROS was similar to wild type.

Mass spectrometry-based proteomics is the method of choice to determine the consequences of modulated protease activity at a molecular level (Demir *et al.*, 2018). Quantitative proteomic analysis of our transgenic *FTSH12* lines revealed large changes in the total protein extract, with >45% of all quantified proteins exhibiting significant changes in abundance amongst the three lines. In line with the pale-green phenotype, plastid proteins including proteins associated with photosynthesis, chlorophyll biosynthesis and maintenance of the photosynthetic machinery, were depleted in the *mi12-3* line, whereas proteins associated with lytic compartments and catabolic processes accumulated (Supplementary Table S6). In contrast, *ox12-1* contained higher amounts of photosynthesis-related proteins compared with wild type, suggesting accelerated plastid development and higher chlorophyll content during de-etiolation (Fig. 2C). Interestingly, the different FtsH12 motor complex subunits showed differential expression in the mutants (Table 2). FtsHi subunits faithfully reflected FtsH12 abundance and accumulated in the overexpressor line *ox12-1*, but dropped below detection limits in the knock-down line *mi2-3*. As expected for a protein with a major metabolic function, pdNAD-MDH was much more abundant than the other subunits and did not show significant differences between wild type and *mi12-3*, suggesting that only a minor fraction of the total pdNAD-MDH is involved in the moonlighting function in the FtsH12 complex (Schreier *et al.*, 2018). In contrast, YCF2, the only plastid-encoded putative interaction partner of FtsH12, showed a moderately increased abundance in wild type, but no significant difference between *ox12-1* overexpressor and *mi12-3* knock-down line. At the transcript level the expression of *YCF2* followed the transcription patterns of the well-known TIC import machinery (Supplementary Fig. S9). Also, pull-down experiments of proteins cross-linked to translocating model proteins clearly identified all four FtsHi subunits and YCF2, but only trace amounts of FtsH12 (Kikuchi *et al.*, 2018), while we did not detect YCF2 in our immunoprecipitation experiments with Myc-tagged FtsH12 expressed from its native promoter. Interestingly, FtsH12 was recently also found in a complex with FtsHi1,2,4,5 and plastid pdNAD-MDH using a combination of native gel electrophoresis and absolute protein quantification by MS (Schäfer *et al.*, 2019). YCF2 was not detected in this study. Although expressed at low absolute levels, FtsH12 appeared twice as abundant as the FtsHi subunits in the complex (Schäfer *et al.*, 2019). One might therefore speculate that accumulation of the FtsH12/FtsHi complex depends on the amount of FtsH12, possibly in a 1:2 stoichiometry of FtsH12 to FtsHi, similar to the ratio of A-type and B-type FtsH proteases in the thylakoid FtsH complex (Kato and Sakamoto, 2018), whereas YCF2 accumulates as a stable protein, independent of FtsH12. However, further experiments are required to determine the stoichiometry of the complex and answer the question as to whether YCF2 is indeed absent from the FtsH12/FtsHi/pdNAD-MDH complex at native expression levels, or if we lost the protein due to the chosen buffer and wash conditions.

Based on the proposed function of the FtsH12 complex as an import motor (Kikuchi *et al.*, 2018), the phenotype of *FTSH12* knock-down might limit protein import efficiency via the 1 MDa TIC complex. The accumulation of TIC20/56/100/214 subunits in the knock-down mutant compared with wild type (Table 2) might be explained by a partial compensation of the limited import capacity by an increase in the number of TIC complexes, possibly in conjunction with the association of an alternative motor complex. Although most other identified TIC components showed no significant changes, TIC22-III accumulated in the knock-down mutant compared with wild type. This may further indicate a modified import complex composition to compensate for the lack of FtsH12, as TIC22-III has been suggested to participate in chloroplast protein import when the required import rate is as high as during early development (Rudolf *et al.*, 2013). Unfortunately we were not able to isolate intact chloroplasts from the *mi12* knock-down mutants, and therefore could not test this attractive hypothesis with import experiments.

Compared with other active FtsH proteases, FtsH12 contains several amino acid exchanges in the Walker B motif and the second region of homology of its AAA-type domain that likely impair the ATPase activity function. In canonical FtsH proteases, ATPase activity is required for extraction and presentation of membrane protein substrates to the protease domain (Bieniossek *et al.*, 2009). Thus, formation of a complex with the proteolytically inactive FtsHi members may be required to enable proteolytic activity of FtsH. Assuming a degradative function of FtsH12, proteins accumulating in the miRNA mutant compared with wild type and/or the overexpression line (clusters 2 and 5; Fig. 5) that are located in the chloroplast envelope appear as the most likely candidate substrates. However, this cluster included few envelope proteins, and most of these, such as the fatty acid transporter FAX1 and components of the chloroplast import machinery that were reported as general features of albino/pale-green mutants, mitigate the lack of protein import capacity and photosynthesis (Motohashi *et al.*, 2012). Also the increased protein abundance of the TIC components accumulating in *mi12-3* appears to arise from transcriptional changes (Supplementary Fig. S9) and not from lack of FtsH12-mediated degradation. In addition, a proteolytically inactive FtsH12 variant has been reported to complement an embryo-lethal T-DNA insertion line, demonstrating that the protease activity was not required for its essential function (Kikuchi *et al.*, 2018). We hypothesized that FtsH12 might affect protein processing either directly by its proteolytic activity or indirectly through impairment of normal proteolytic maturation after import. However, our N-terminome analysis revealed overall good correlation of protein N-termini abundance and protein abundance. The few protein N-termini with strong changes in abundance and unchanged protein abundance were manually inspected, but did not include protease-generated termini of plastid proteins, and therefore, likely represent indirect effects arising from differential activation of other proteolytic activities, as for example lytic enzymes in the vacuole. We further hypothesized that impaired protein import due to lack of FtsH12 might lead to a backlog of unprocessed precursor proteins, and indeed identified 12 N-terminal peptides of nuclear-encoded plastid proteins that mapped to position 2 of the protein model, indicating intact chloroplast targeting peptides (Supplementary Table S7). Eleven of these did not contain a Lys that would allow quantification in our assay, while the remaining precursor of an uncharacterized protein encoded by gene At4g28440 showed no significant difference in accumulation. This was not unexpected, as unprocessed precursor proteins accumulating in the cytosol are rapidly degraded by the ubiquitin/proteasome system (Lee *et al.*, 2009; Grimmer *et al.*, 2020).

In conclusion our data confirm the existence of a heteromeric FtsH12 complex with FtsHi1, FtsHi2, FtsHi4, FtsHi5 and pdNAD-MDH in the inner chloroplast envelope membrane (Kikuchi *et al.*, 2018; Schreier *et al.*, 2018; Schäfer *et al.*, 2019). Plants with modulated expression of *FtsH12* showed dramatic changes in proteome composition, and demonstrate that the abundance of FtsH12 regulates the accumulation of the FtsHi subunits, but not pdNAD-MDH or YCF2. Plants with reduced *FTSH12* expression were impaired in the formation of thylakoid membranes, whereas *FTSH12* overexpressing plants appeared to develop slightly faster. N-terminome profiling demonstrated unaltered maturation of chloroplast-imported proteins, irrespective of the abundance of FtsH12.

## Supplementary data

The following supplementary data are available at *JXB* online.

Fig. S1. Domain structure of FtsH12.

Fig. S2. Expression of *FTSH12* in different organs of *A. thaliana* (Col-0) and during different developmental stages.

Fig. S3. Expression of *FtsH12* during development.

Fig. S4. Sub-organellar location of FtsH12 in chloroplasts.

Fig. S5. Identification of homozygous T2 FtsH12-4x-c-myc T-DNA insertion into *FTSH12*.

Fig. S6. Analysis of co-immunoprecipitation experiments.

Fig. S7. Phenotypic comparison of wild type, *FtsH12* overexpressor and knock-down plants.

Fig. S8. Occurrence of reactive oxygen species in wild type and *FTSH12* transgenic lines.

Fig. S9. Transcript abundance of import complex subunits.

Fig. S10. Graphical depiction of protein interactions in Cluster 1 of the proteome analysis.

Fig. S11. Graphical depiction of protein interactions in Cluster 2 of the proteome analysis.

Fig. S12. Graphical depiction of protein interactions in Cluster 3 of the proteome analysis.

Fig. S13. Graphical depiction of protein interactions in Cluster 4 of the proteome analysis.

Fig. S14. Graphical depiction of protein interactions in Cluster 5 of the proteome analysis.

Fig. S15. Graphical depiction of protein interactions in Cluster 6 of the proteome analysis.

Fig. S16. Graphical depiction of protein interactions in Cluster 7 of the proteome analysis.

Fig. S17. Quantitative N-terminome analysis in *FtsH12* mutants compared with wild type.

Table S1. Sequences of primers used in this study.

Table S2. List of proteins identified my mass spectrometry after FtsH12:Myc immunoprecipitation

Table S3. Densitometric analysis of FtsH12 protein amounts (% of wild type) in 10 day-old *ftshi* seedlings.

Table S4. Chlorophyll fluorescence parameters.

Table S5. List of 3129 quantified proteins.

Table S6. List of 1450 proteins with significantly changed abundance between *mi12-3*, *ox12-1* and wild type, as determined by ANOVA analysis.

Table S7. List of identified protein N-termini with quantification result and annotation.

eraa550_suppl_Supplementary-Figures-S1-S17_and_Tables-S1-S3-S4

eraa550_suppl_Supplementary-Tables-S2-S5-S7

## Data Availability

MS data are available on the PRIDE archive (https://www.ebi.ac.uk/pride/archive/; Vizcaíno *et al.*, 2015; Deutsch *et al.*, 2017) with the accession numbers PXD010619 for proteome, PXD022423 for N-terminome and PXD010615 for AP-MS data. Novel materials described in this paper will be made available for non-commercial research purposes.
