# Supplementary material for: Abundance of metalloprotease FtsH12 modulates chloroplast development in Arabidopsis thaliana
Source: J Exp Bot. 2020 Nov 20;72(9):3455–73. doi: 10.1093/jxb/eraa550 (PMC8042743; doi:10.1093/jxb/eraa550)
Supplement: eraa550_suppl_Supplementary-Figures-S1-S17_and_Tables-S1-S3-S4 [file eraa550_suppl_supplementary-figures-s1-s17_and_tables-s1-s3-s4.pdf]

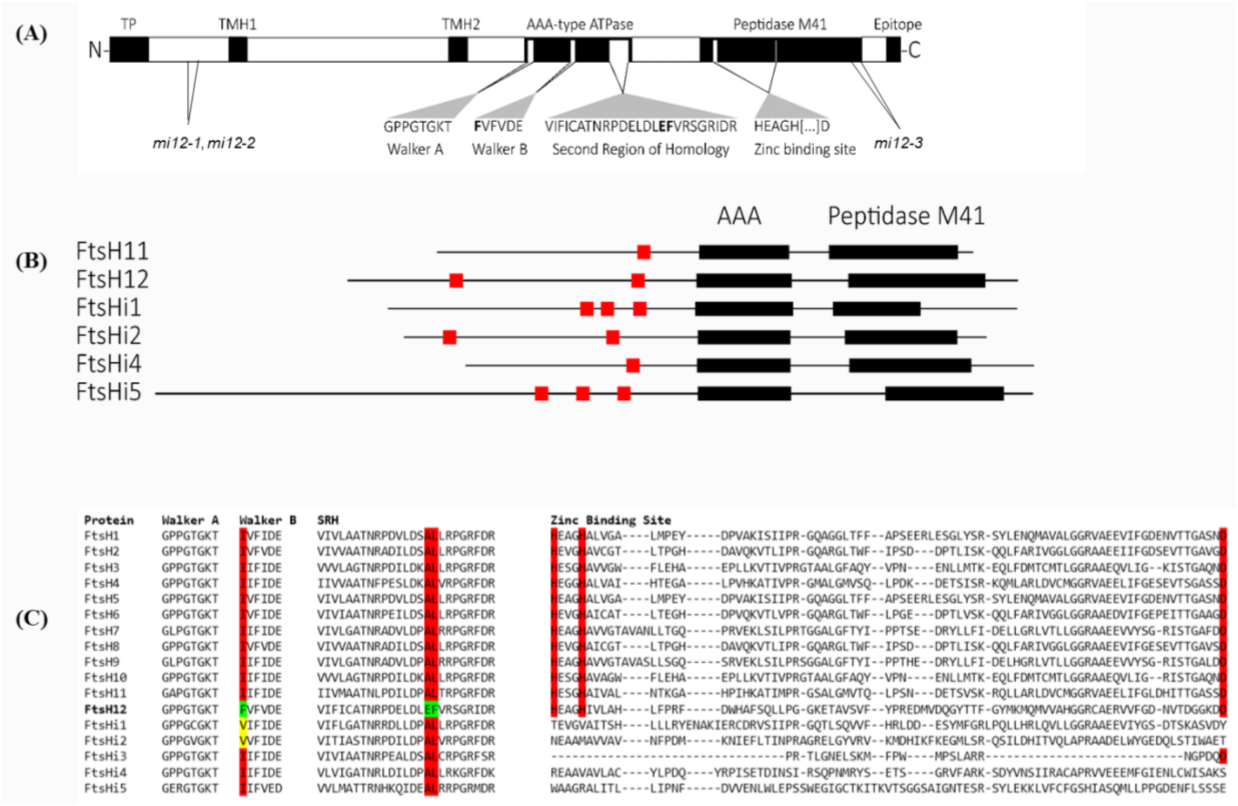

Fig. S1: Domain structure of FtsH12. (**A**) Domains are shown as black boxes; certain amino acid sequences are given below. FtsH12 contains an N-terminal transit peptide directing it into plastids (TP), start of the mature protein is shown below based on identified N-termini, two transmembrane helices (TMH1 and TMH2), an AAA-type domain (AAA-type ATPase) and the proteolytic domain (Peptidase M41). Changes of conserved amino acids are indicated as bold letters. *mi12-1*, *mi12-2* and *mi12-3* indicate the region of mutation of the micro-RNA lines. (**B**) Scale models of domains of the FtsH12-FtsHi complex and FtsH11 as comparison. Scaling is indicated by a 100 amino acid piece below all models. Black lines represent primary amino acid sequence of the corresponding FtsH member, red boxes represent predicted trans-membrane helices (UNIPROT), and black boxes represent the AAA-like and the peptidase M41 domains given by InterProScan. (**C**) Sequence alignments of motives common for the FtsH protein family of *A. thaliana*. Conserved amino acids are marked with red background, green background marks amino acid changed in FtsH12, while yellow background shows changes in FtsHi.

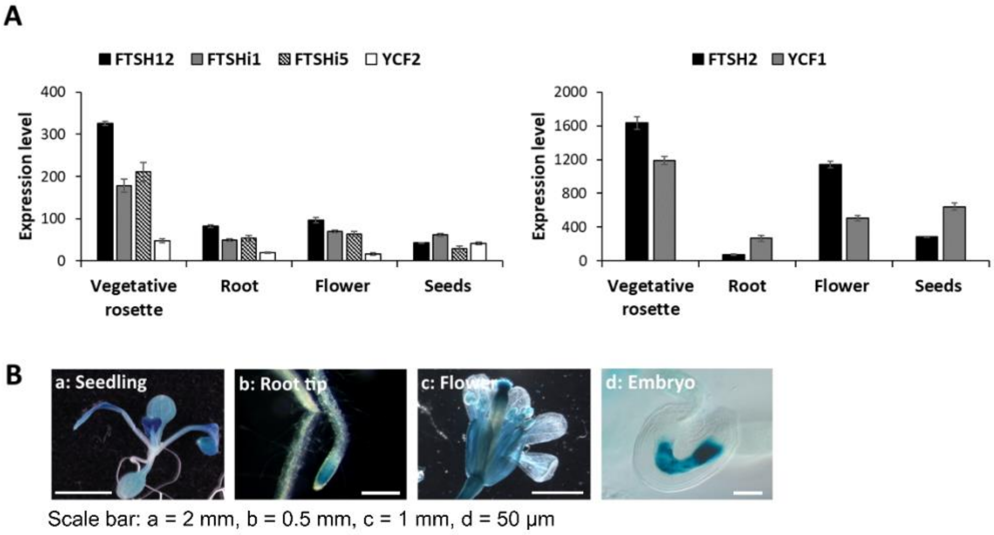

Fig. S2: Expression of *FTSH12* in different organs of *A. thaliana* (Col-0) and during different developmental stages. (**A)** Expression in leaves, roots, flowers and seeds of *FTSH12* and genes encoding other subunits of the FtsH12/FtsHi complex (*FTSHi1*, *FTSHi5*, *YCF2.2* (ATCG01280), left panel) as well as genes encoding the thylakoid located FTSH2 and the import complex subunit YCF1.1 (ATCG0100) (right level) were extracted from the Arabidopsis eFP browser (http://bar.utoronto.ca/). Data were normalized by the GCOS method, TGT value of 100 (Winter *et. al.*, 2007). (**B**) *FTSH12* expression in transgenic Arabidopsis T3-lines containing the native *FTSH12* promoter fused to the β-glucuronidase (GUS) gene. GUS activity was made visible by histochemical staining in leaves (**Ba**) and roots (**Bb**) of 12-day old plants, flowers (**Bc**), and at embryo stage (**Bd**).

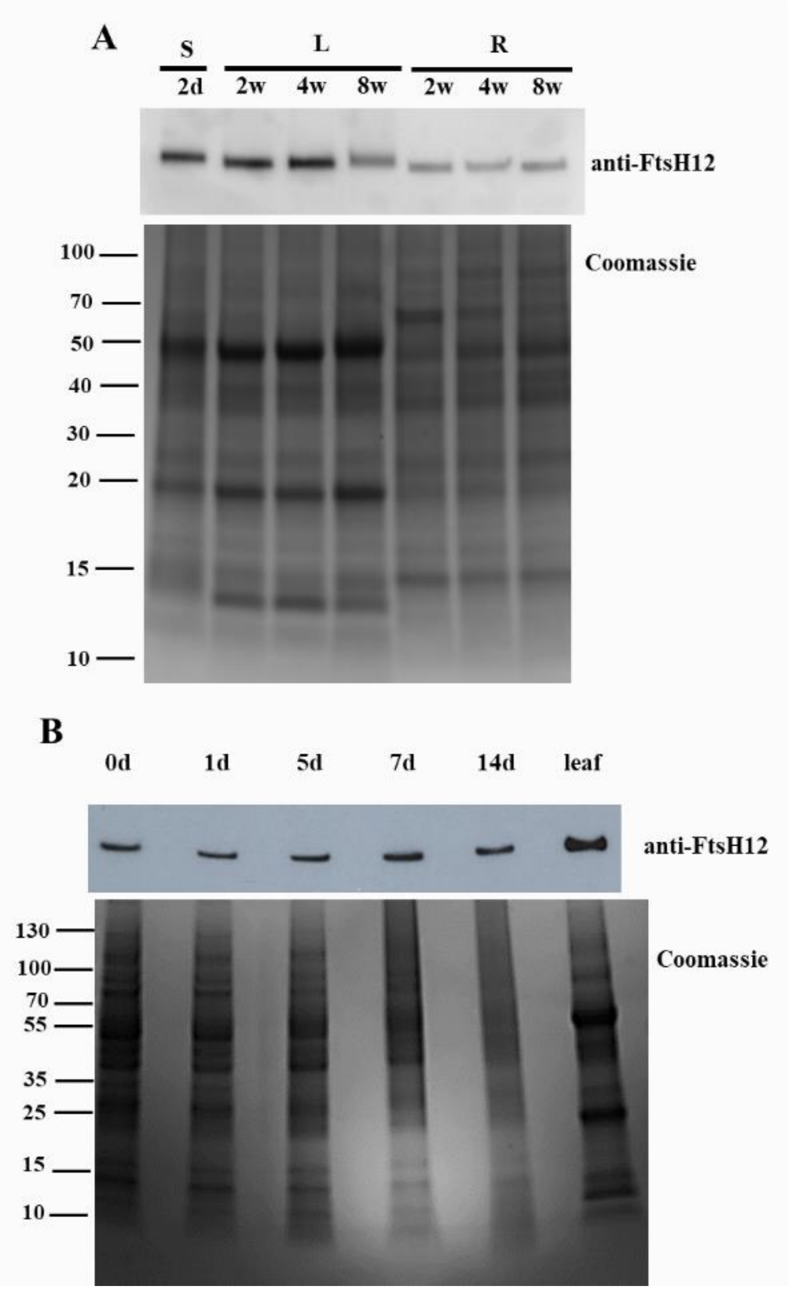

Fig. S3: Expression of FtsH12 during development. Immunostain of FtsH12 protein amount in different organs and developmental stages (**A**) and during chloroplast development in an *A. thaliana* cell culture (**B**). **A**: S-seedlings, L-leaves, R-roots, d-days, w-weeks. Seedlings and two weeks old plants were grown on MS-medium growth plates, while four and eight weeks old plants were grown on soil. **B**: Cells cultivated in darkness contain proplastids (0 d). On illumination chloroplasts start to develop (day 1), thylakoid structures are established at day 5 and are able to perform photosynthesis at day 7. Cells being illuminated for 14 days contain mature chloroplasts

(Dubreuil *et al.*, 2018). 15 µg of protein were loaded onto the SDS-PAGE, three biological replicates were tested.

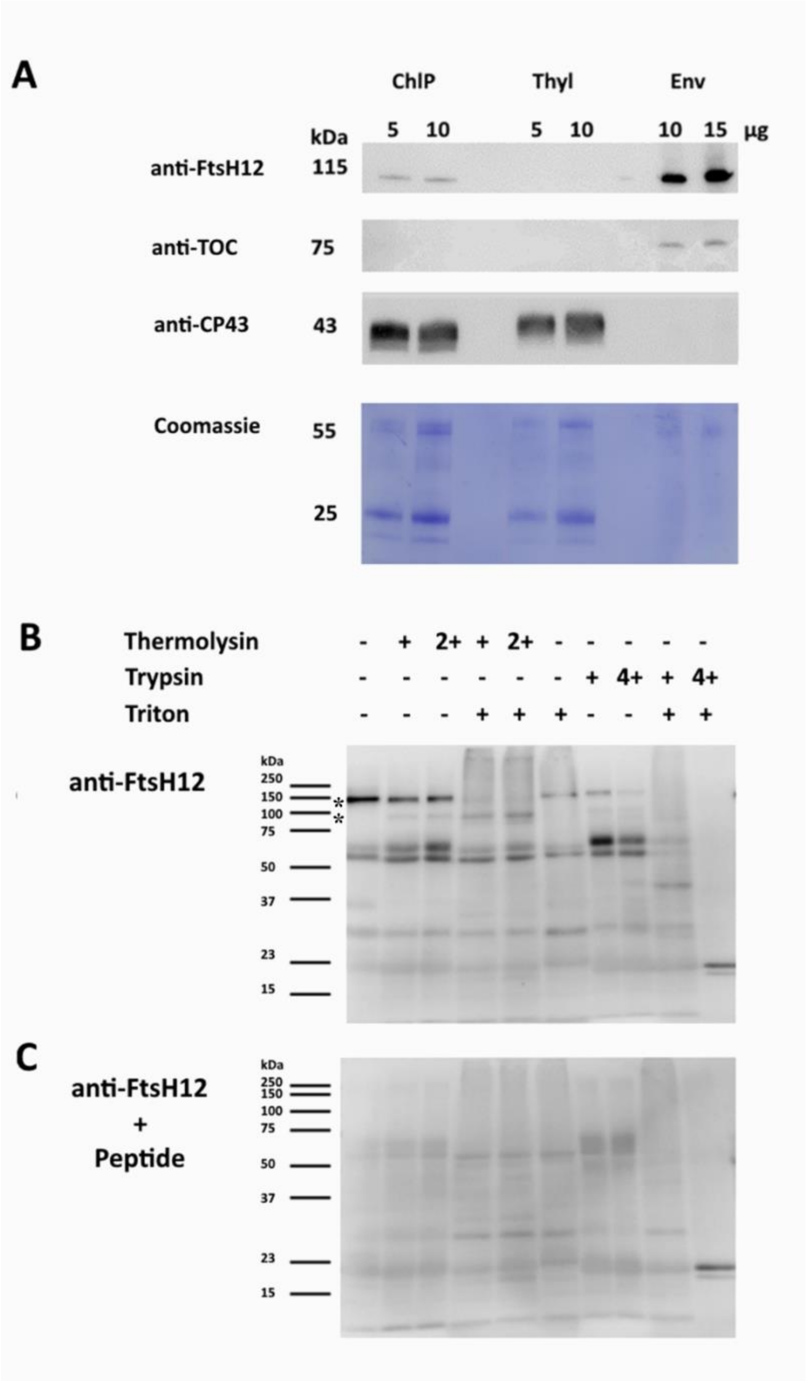

Fig. S4: Sub-organellar location of FtsH12 in chloroplasts. (**A**) Immunological staining of FtsH12 in whole chloroplasts (ChlP), thylakoid membranes (Thyl) and the chloroplast envelope (Env) isolated from four-weeks-old wild type plants. Antibodies directed against the envelope marker TOC75 and thylakoid marker CP43 were used as control. A representative of three biological replicates is shown. (**B**) For an i*n organello* protease protection assay purified

chloroplasts were treated with thermolysin or trypsin in the presence or absence of 2 % Triton X-100 at concentrations of 100 µg/ml (+), 200 µg/ml (2+), or 400 µg/ml (4+). Each lane was loaded with 15 µg protein and immunostained using the anti-FtsH12 antibody. (**C**) Peptide quenching using the FtsH12 peptide antigene was performed as a control of the immunostaining. Specific immunobands present only in (B) belong to FtsH12 and are marked by asterisk.

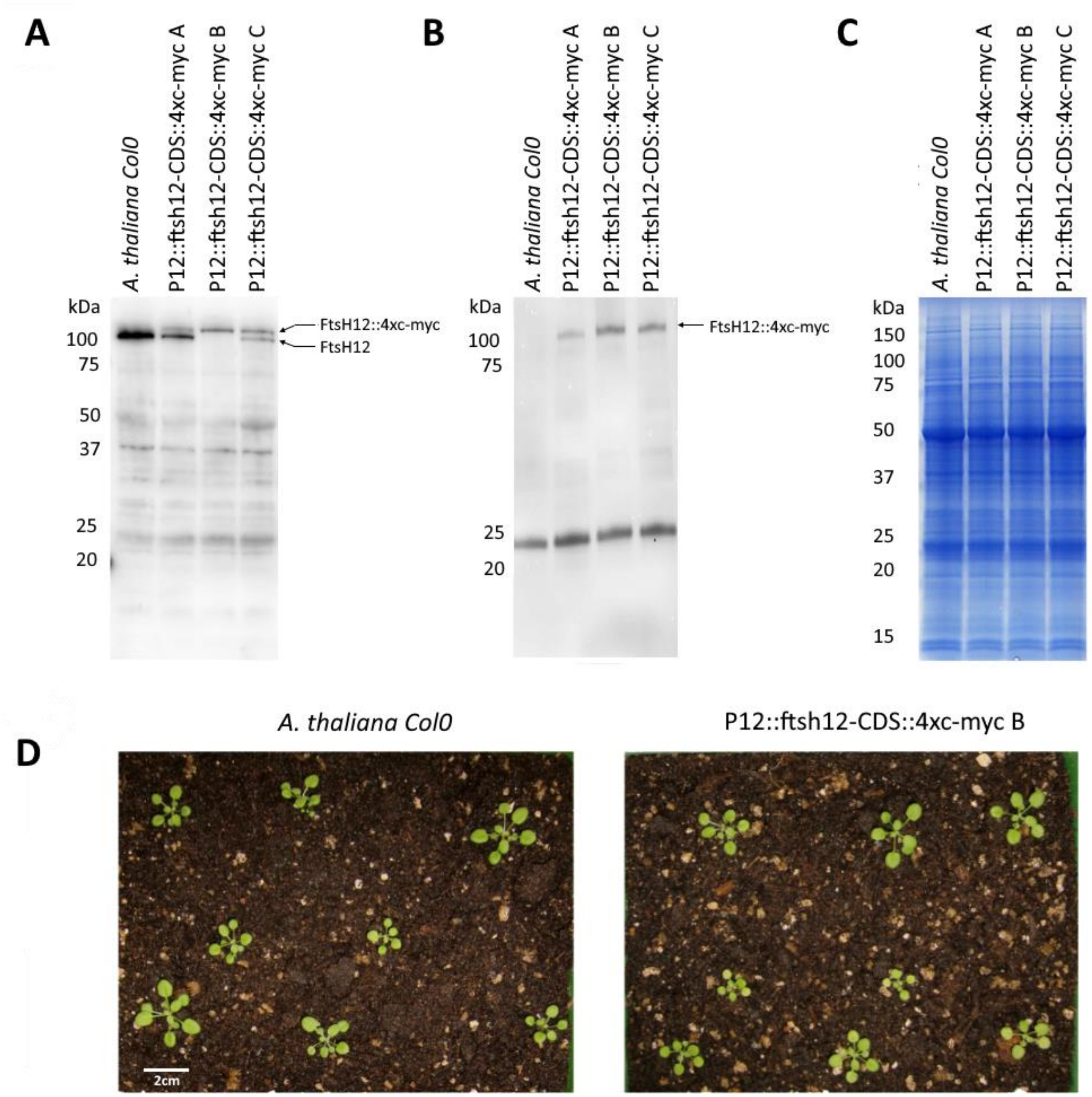

Fig. S5: Identification of homozygous T2 FtsH12-4x-c-myc T-DNA insertion into *FTSH12*. Plants of the heterozygote *FTSH*/*ftsh*12 T-DNA insertion line (GABI_550G09) were transformed with a promoter-*ftsh*12::*ftsh*12CDS::4xC-myc construct and segregated. (**A**) Immunoanalysis of total leaf protein extracts using the anti-FtsH12 antibody. The lower band corresponds to wt FtsH12 (112 kDa, lane 2), while the upper band represents c-myc tagged FtsH12. Line B is homozygous for the T-DNA insertion and lacks native FtsH12. (**B**) Immunoanalysis of total leaf

protein extracts using the anti-myc antibody. (**C**) Coomassie-blue stained SDS PAGE showing the protein pattern as loading control. (**D**) Seedling phenotype of the P12::ftsh12-CDS::4xc-myc B line (right panel) in comparison to wild type (left panel).

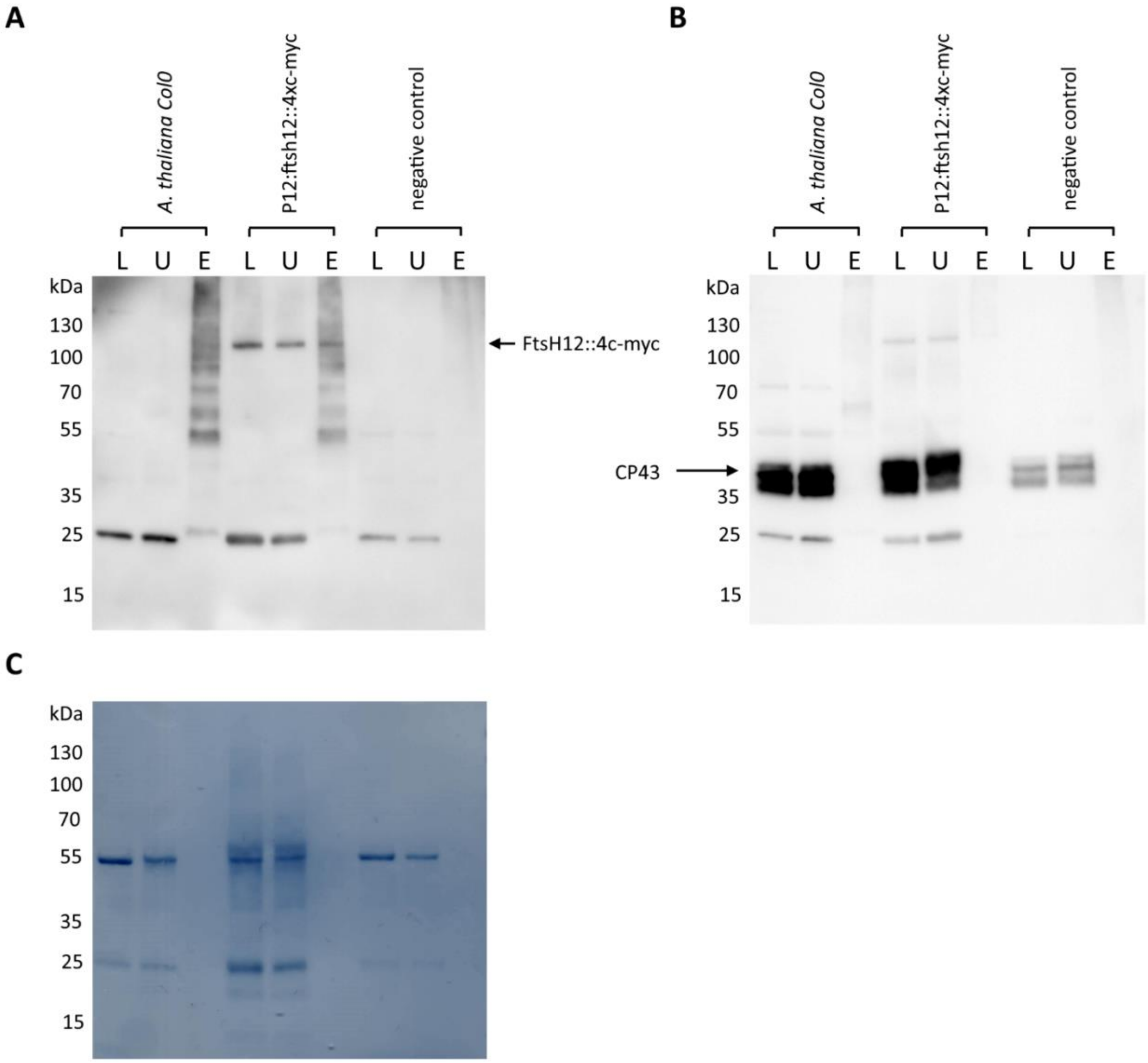

Fig. S6: Analysis of co-immunoprecipitation experiment. Chloroplasts isolated from 6-weeks-old wt-and homozygous P12::ftsh12:4xc-myc plants were used for co-immunoprecipitation. Samples collected during Co-IP were immunoblotted and analysed using first an anti-myc (**A**) and after stripping an anti-CP43 antibody (**B**). L= solubilized chloroplast proteins before addition of antibody-coated beads; U = unbound proteins (flow through); E = eluate. Beads coated with anti-his antibody instead of anti-myc antibody were used as negative control. (**C**) Membrane stained with Coomassie**.**

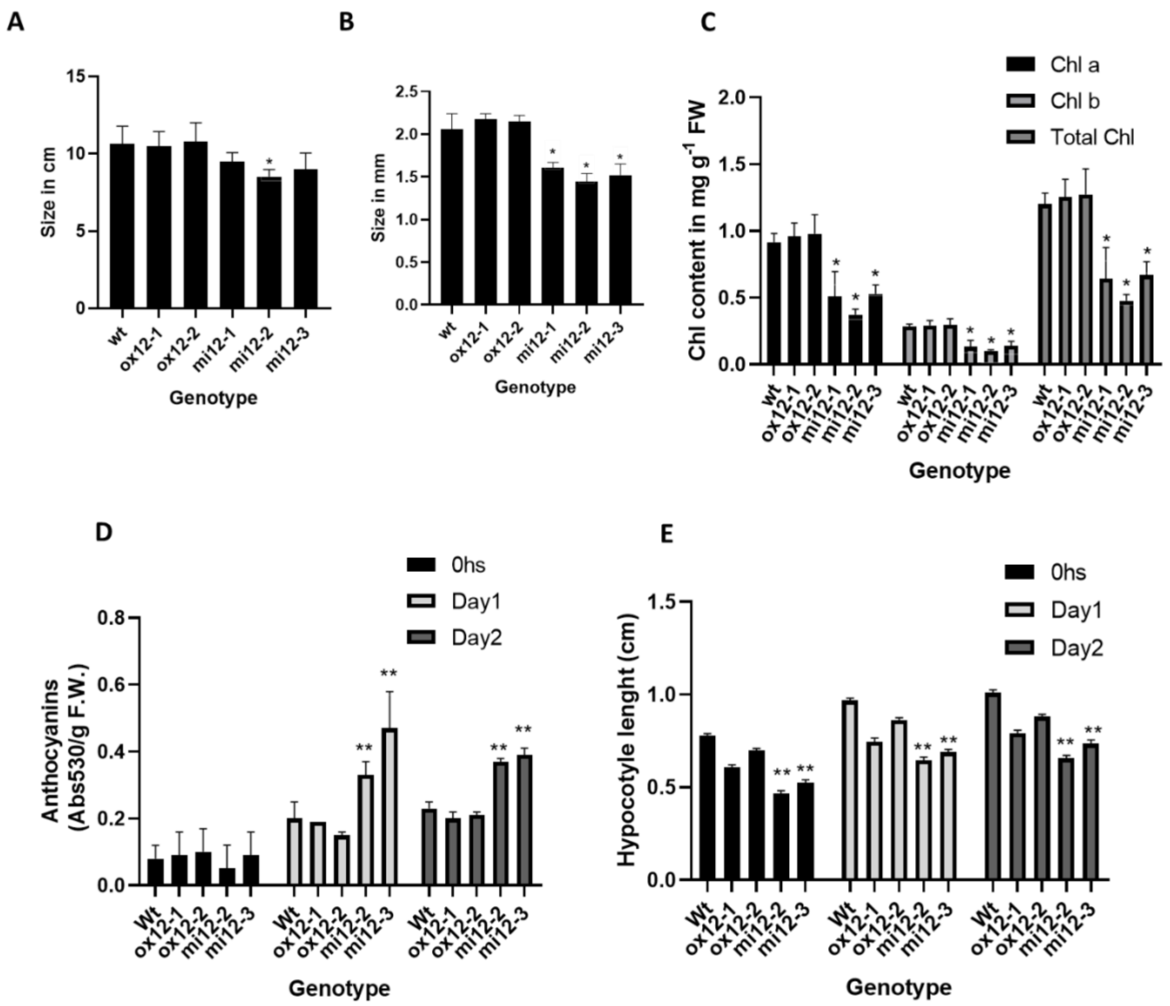
A
Size in cm
Genotype
wt
ox12-1
ox12-2
mi12-1
mi12-2
mi12-3
B
Size in mm
Genotype
C
Chl a
Chl b
Total Chl
Chl content in mg g-1 FW
Genotype
D
0hs
Day1
Day2
Anthocyanins (Abs530/g F.W.)
Genotype
Wt
E
Hypocotyle lenght (cm)
Genotype

Fig. S7 Phenotypic comparison of wild type, *FtsH12* overexpressor and knock-down plants. **.** (**A**) Rosette diameters of 9-week-old plants and (**B**) tip-to-tip sizes of cotyledons in two-day old seedlings comparing wt, the FtsH12 overexpressor (*ox12*) and knock-down (*mi12*) lines. Values are averages (± SE) of three independent experimental replicates with 8 to 20 plants per single experiment. Significant differences between wt and transgenic plants are indicated by asterisk (Student's t-test, $p < 0.05$).

(**C**) Chlorophyll amount (mg g^{-1} fresh weight) in developing leaves of 10 weeks-old wt and *FTSH12* transgenic plants. Values are averages (± SE) of three independent experimental replicates, each with three plants per experiment (Student's t-test, $p < 0.05$).

(**D**) Quantification of anthocyanin (Abs530/g fresh weight) of wt and transgenic *FTSH12* seedlings during de-etiolation.

(**E**) Hypocotyl length of wt and transgenic *FTSH12* seedlings during de-etiolation. Values represent the mean ± se of measurements conducted on $n = 75$, $n = 71$ and $n = 73$ seedlings for wt, overexpressor and knock-down lines, respectively. Significant differences between wt and transgenic plants are indicated by asterisk (Student's t-test, $p < 0.05$).

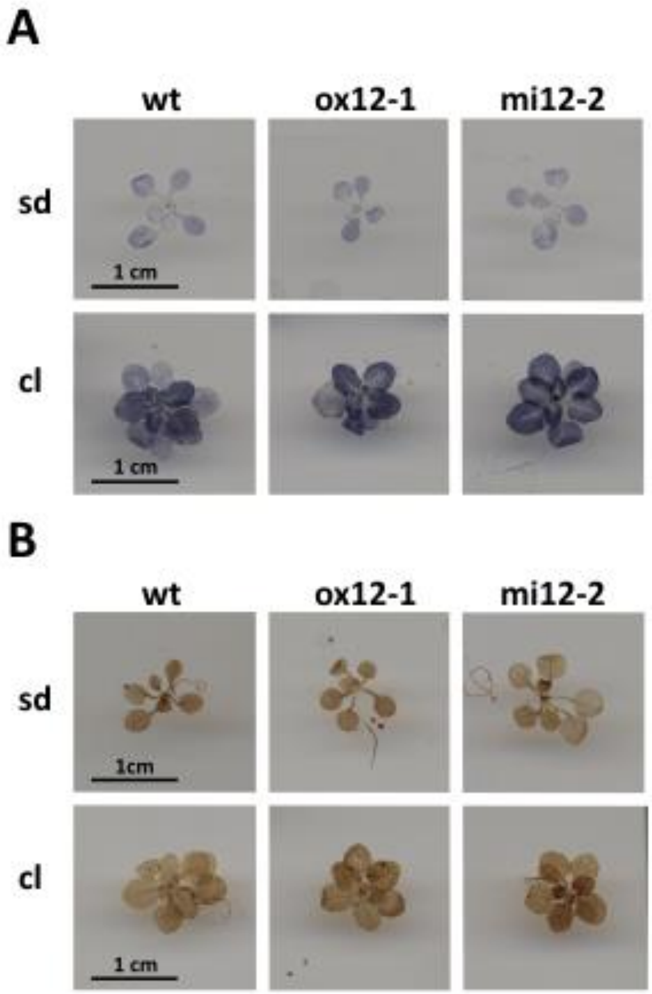

Fig. S8: Occurrence of reactive oxygen species in wild type and *FTSH12* transgenic lines. (**A**) Histochemical superoxide and (**B**) hydrogen peroxide staining of 10-days-old seedlings grown at short day conditions (sd) or continuous light (cl).

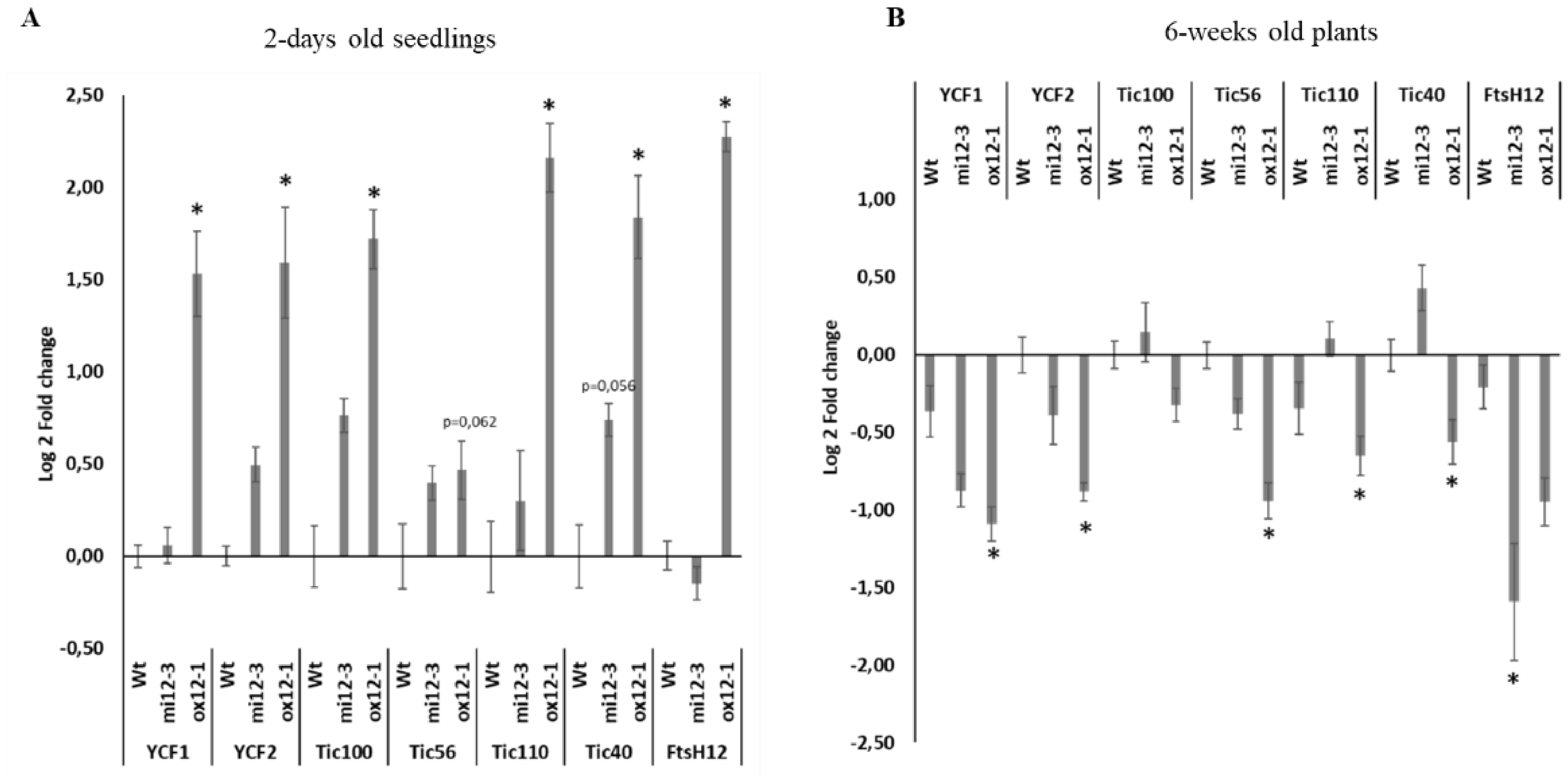

Fig. S9. Transcript abundance of import complex subunits. qPCR was performed on RNA extracted from 2-days old seedlings (**A**) or 6-weeks old plants (**B**) of wild type, the *FTSH12* knock-down line *mi12-3* and the *FTSH12* overexpressor line *ox12-1*. Significant differences between wt and the transgenic lines are indicated by asterisk (one way Anova LSD post-hoc test, $p < 0.05$).

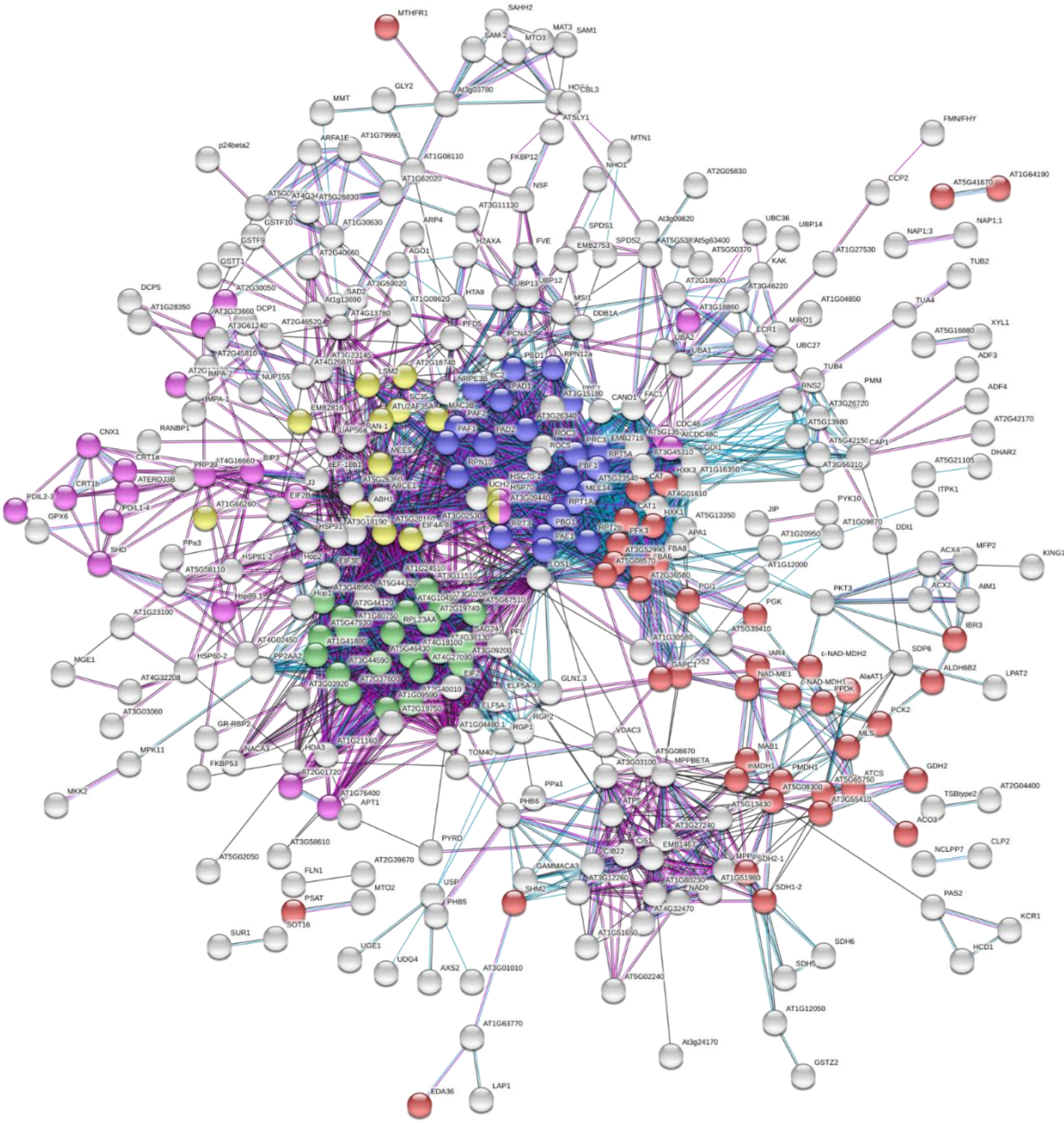

Fig. S10. Graphical depiction of protein interactions in Cluster 1 of the proteome analysis. KEGG functional term enrichment, protein interactions and protein coexpression were analysed using the STRING database. Blue edges indicates proteins assocations annotated in databases, pink from experimental evidence, black indicates coexpression. Enriched KEGG pathways are indicated: red, ath01200, carbon metabolism; blue, ath03050, proteasome; green, at03010, ribosome; yellow, ath03040, spliceosome; pink, ath04141, protein processing in the endoplasmic reticulum.

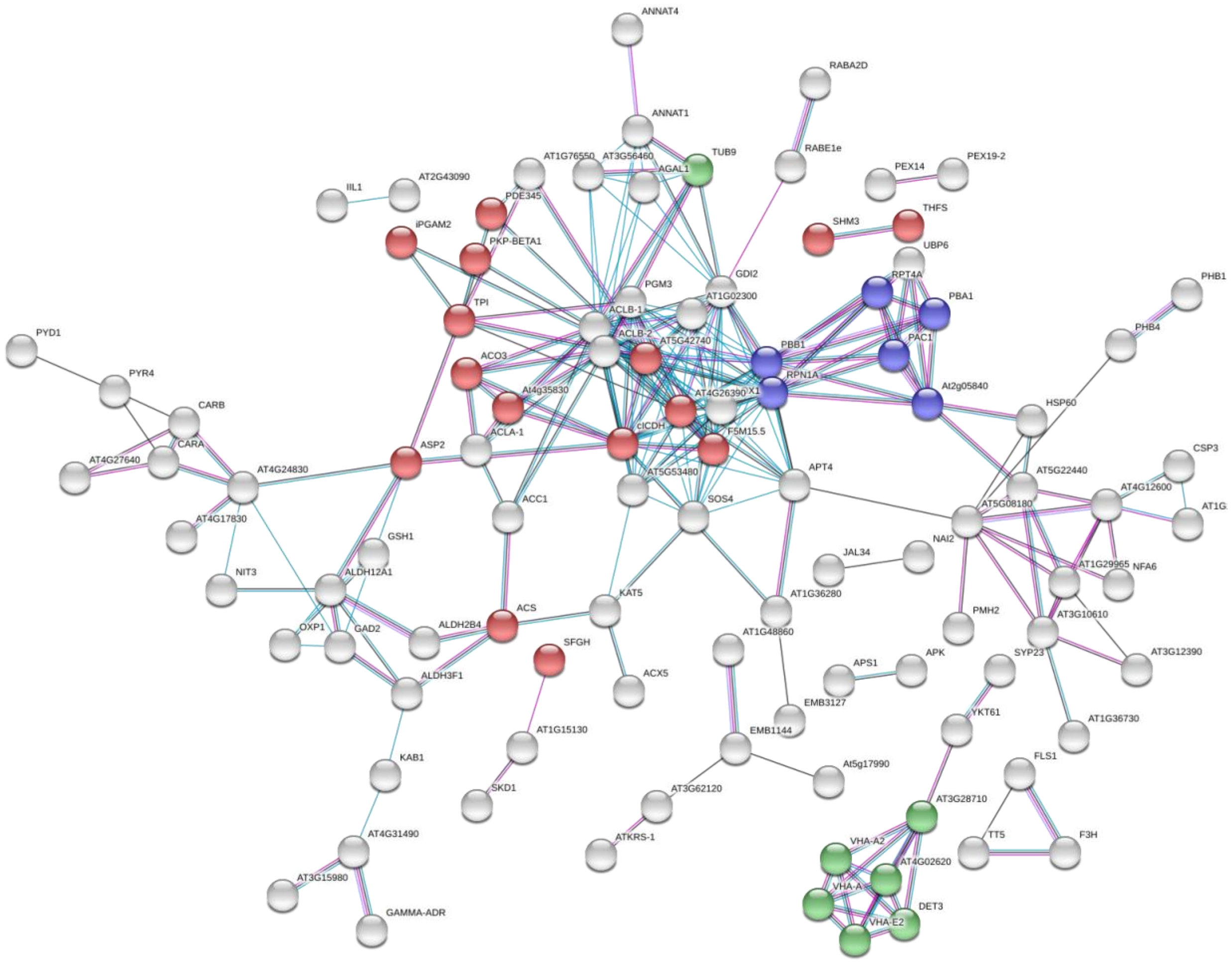

Fig. S11. Graphical depiction of protein interactions in Cluster 2 of the proteome analysis. KEGG functional term enrichment, protein interactions and protein coexpression were analysed using the STRING database. Blue edges indicates proteins associations annotated in databases, pink from experimental evidence, black indicates coexpression. Enriched KEGG pathways are indicated: red, ath01200, carbon metabolism; blue, ath03050, proteasome; green, ath04145, phagosome.

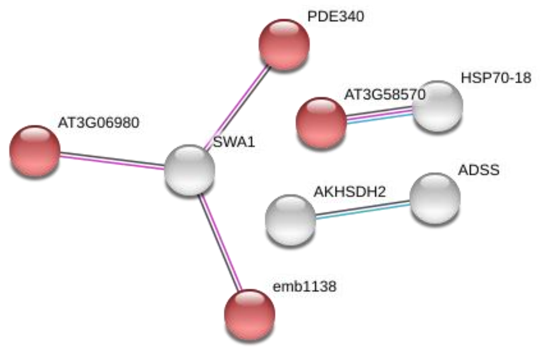

Fig. S12. Graphical depiction of protein interactions in Cluster 3 of the proteome analysis. Functional GO term enrichment was analysed using the STRING database. Blue edges indicates proteins associations annotated in databases, pink from experimental evidence, black indicates coexpression. Red, GO:0004004, ATP-dependent RNA helicase activity.

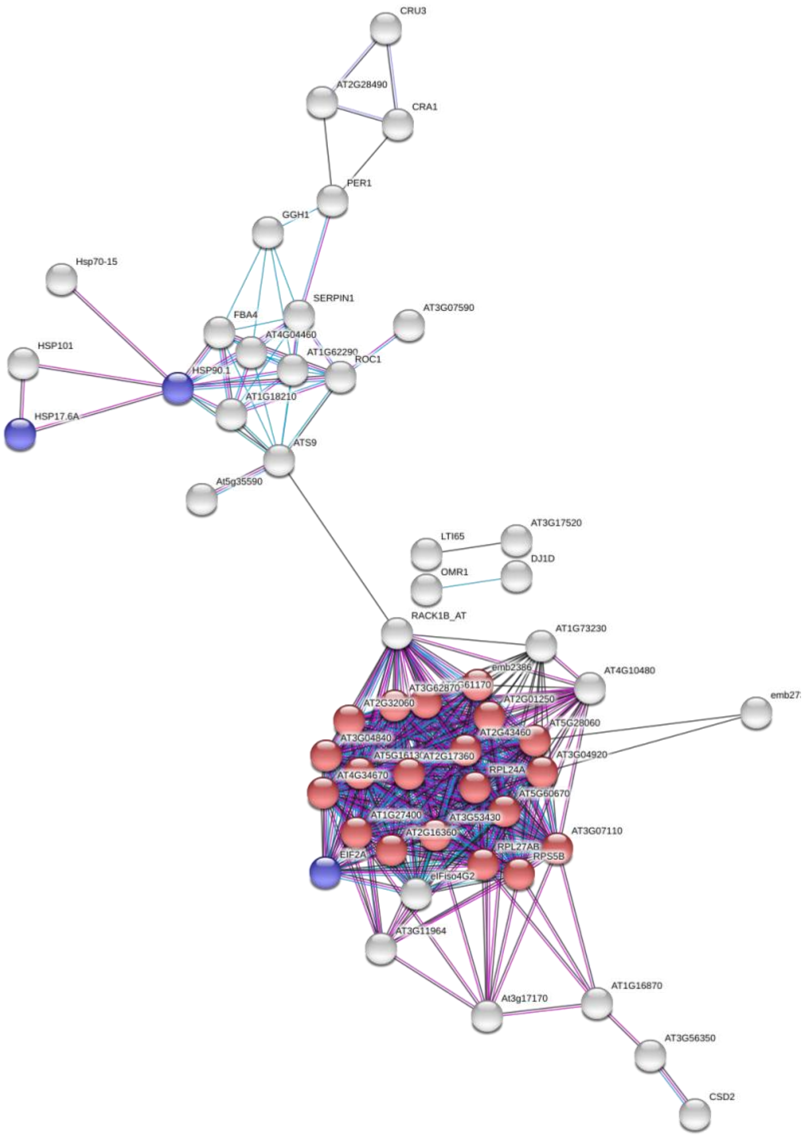

Fig. S13. Graphical depiction of protein interactions in Cluster 4 of the proteome analysis. KEGG functional term enrichment, protein interactions and protein coexpression were analysed using the STRING database. Blue edges indicates proteins associations annotated in databases, pink from experimental evidence, black indicates coexpression. Enriched KEGG pathways are indicated: red, at03010, ribosome; blue, ath04141, protein processing in the endoplasmic reticulum.

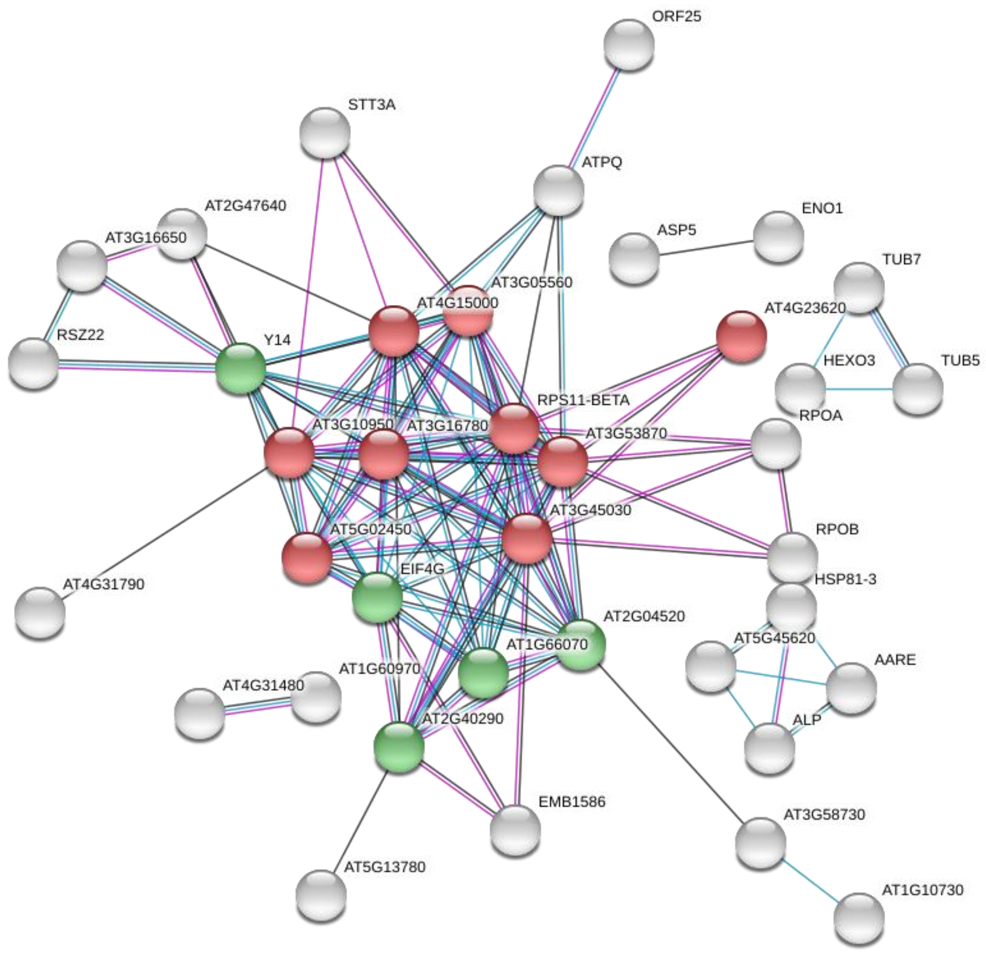

Fig. S14. Graphical depiction of protein interactions in Cluster 5 of the proteome analysis. KEGG functional term enrichment, protein interactions and protein coexpression were analysed using the STRING database. Blue edges indicates proteins associations annotated in databases, pink from experimental evidence, black indicates coexpression. Enriched KEGG pathways are indicated: red, at03010, ribosome; green, ath03013, RNA transport.

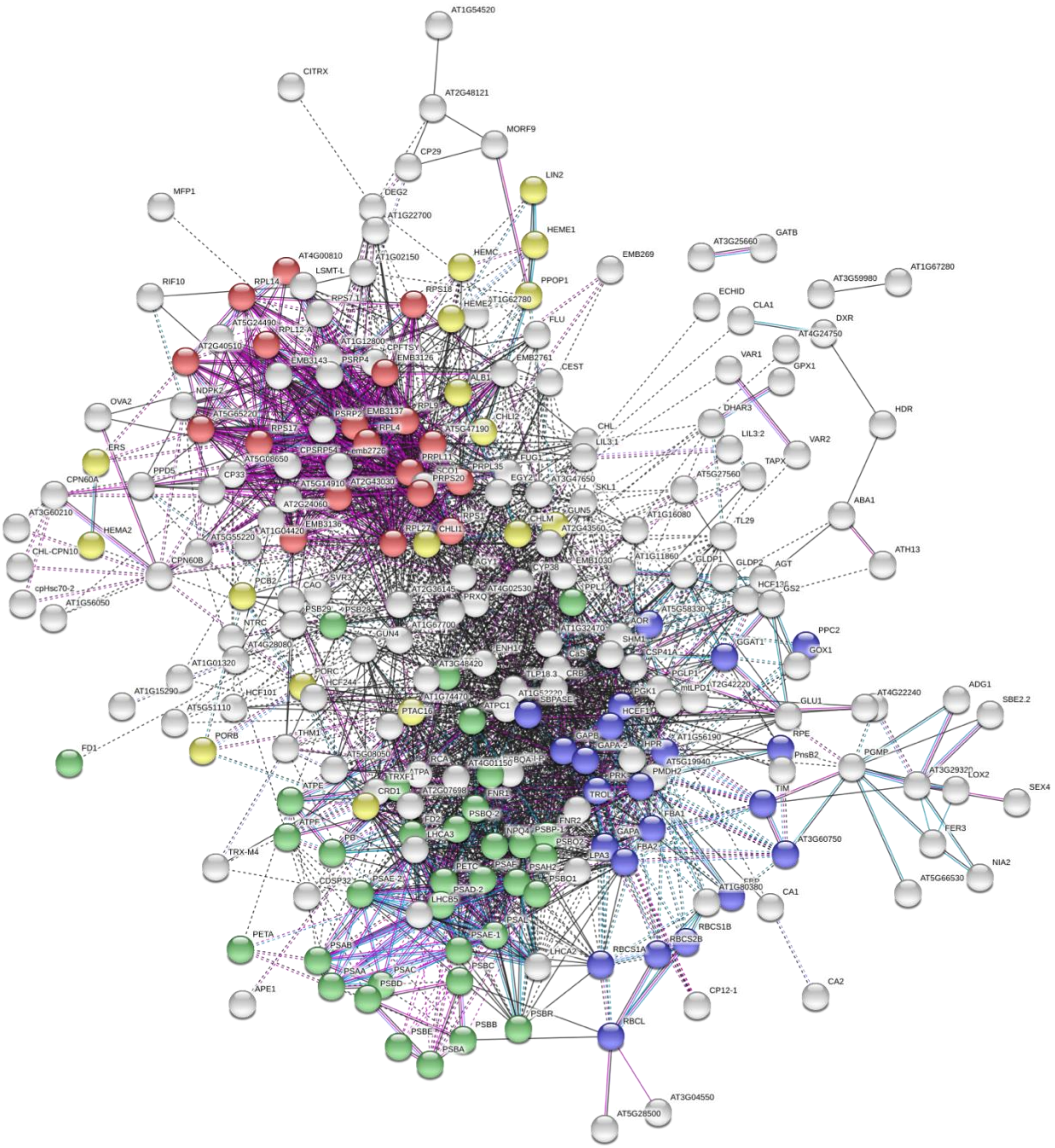

Fig. S15. Graphical depiction of protein interactions in Cluster 6 of the proteome analysis. KEGG functional term enrichment, protein interactions and protein coexpression were analysed using the STRING database. Blue edges indicates proteins associations annotated in databases, pink from experimental evidence, black indicates coexpression. Enriched KEGG pathways are

indicated: red, at03010, ribosome; blue, ath01200, carbon metabolism; green, at00195, photosynthesis; yellow, ath00860, porphyrin and chlorophyll metabolism.

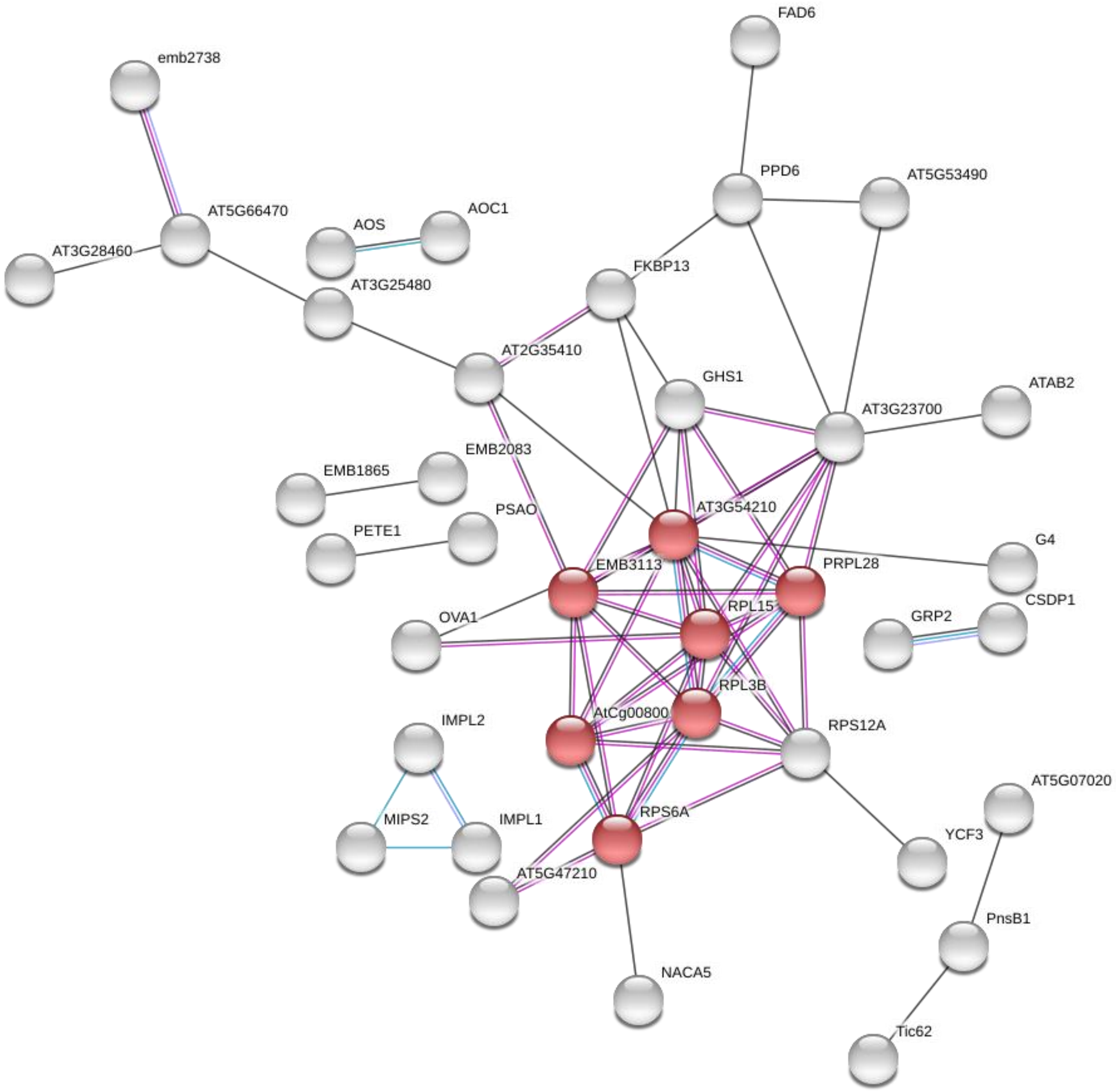

Fig. S16. Graphical depiction of protein interactions in Cluster 7 of the proteome analysis. KEGG functional term enrichment, protein interactions and protein coexpression were analysed using the STRING database. Blue edges indicates proteins associations annotated in databases, pink from experimental evidence, black indicates coexpression. Enriched KEGG pathways are indicated: red, at03010, ribosome.

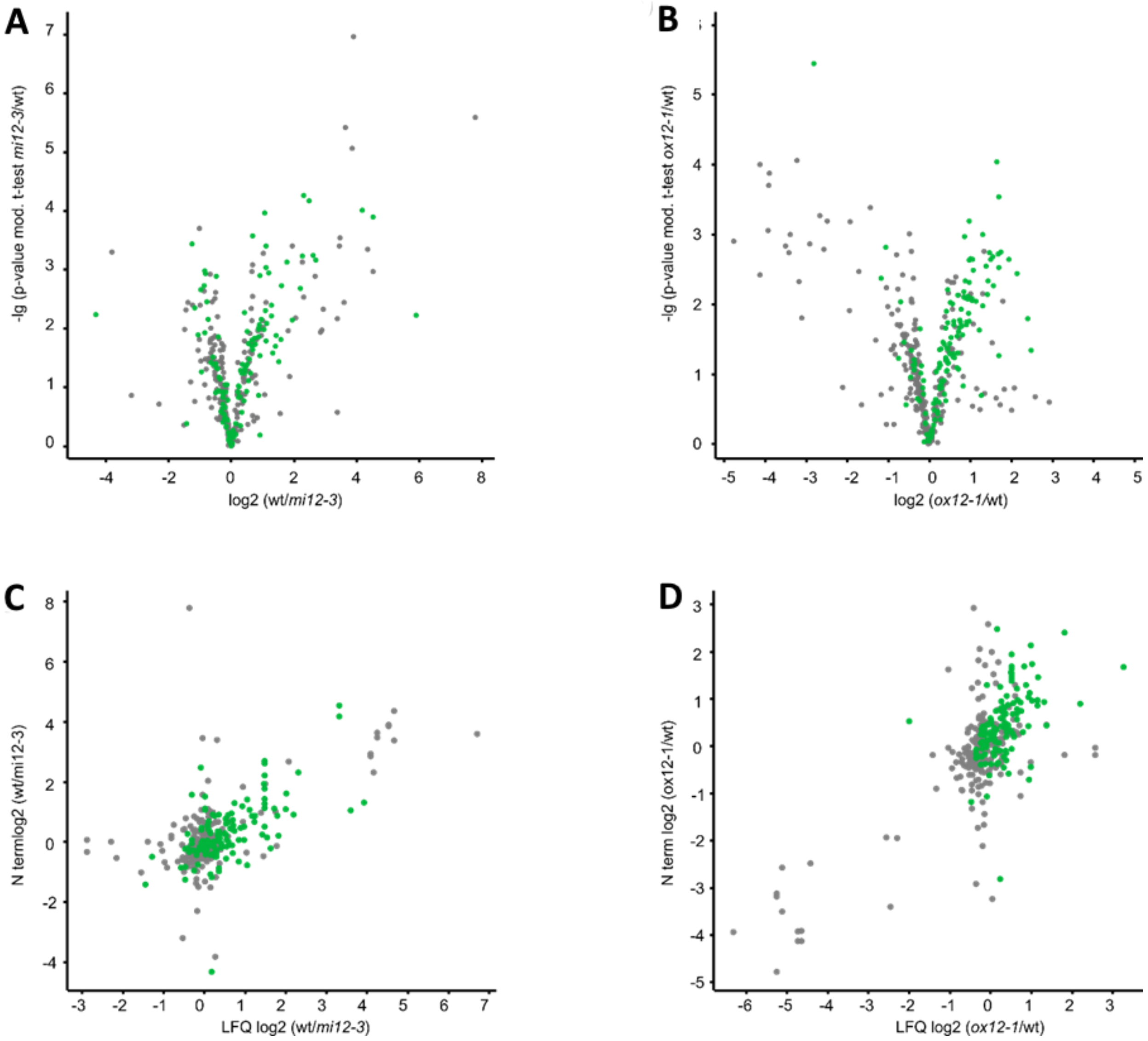

Fig. S17. Quantitative N-terminome analysis in FtsH12 mutants compared with wild-type
Volcano plots show N-terminal peptide abundance in (**A**) wt compared with *mi12-3* and (**B**) *ox12-1* compared with wt. Peptides from plastid-located proteins (plastid encoded or predicted to target to the plastid by TargetP2.0) are highlighted in green. Scatter blots show the correlation with the corresponding protein abundance in (**C**) wt compared with *mi12-3* and (**D**) *ox12-1* compared with wt as determined by label-free quantification.

Supplementary Table S1: Sequences of primers used in this study.

Primer	Sequence (5’ -> 3’)
mi12-1 & mi12-2 I miR-s	gaTAAGGCGAATAGACTGACCTAtctctcttttgtattcc
mi12-1 & mi12-2 II miR-a	gaTAGGTCAGTCTATTCGCCTTAtcaaagagaatcaatga
mi12-1 & mi12-2 III miR*s	gaTAAGTCAGTCTATACGCCTTTtcacaggtcgtgatatg
mi12-1 & mi12-2 IV miR*a	gaAAAGGCGTATAGACTGACTTAtctacatatatattcct
mi12-3 I miR-s	gaTAACACTATATGACGAGCCTGtctctcttttgtattcc
mi12-3 II miR-a	gaCAGGCTCGTCATATAGTGTTAtcaaagagaatcaatga
mi12-3 III miR*s	gaCAAGCTCGTCATAAAGTGTTTtcacaggtcgtgatatg
mi12-3 IV miR*a	gaAAACACTTTATGACGAGCTTGtctacatatatattcct
FtsH12 Forward	caccATGGAGATTGCAATTTCGTATAAAC
FtsH12 Reverse for overexpressor	CTAGCTTCTGTGGAGTGGCGCAGCACG
FtsH12 Promotor Forward	caccGACAACACCCCAAGAAATGTCC
FtsH12 Promotor Reverse	CTTCACAGATTCTCCAATCTTCTC
FtsH12 Reverse for cmyc-line	GCTTCTGTGGAGTGGCG
FtsH12 qF	GAGGAGCTTTTACCTCACAAG
FtsH12 qR	CTGTGGTGTGACAGGATAAG
ACT2-qF	CTTGCACCAAGCAGCATGAA
ACT2-qR	CCGATCCAGACACTGTACTTCCTT
UBQ5-qF	ACGCTTCATCTCGTC
UBQ5-qR	CCACAGGTTGCGTTA
YCF1_qF	CTCAGAAACGGGTGGGACTA
YCF1_qR	GTTCGAACGTGGAATTCAT CATC
YCF2_qF	GAGCCTATAGTGAGTTACAGACA
YCF2_qR	CTATCCAGAAGTTTTCGCAACTC
TIC100_qF	CGAGTGAAGCGTATGCAAGAAG
TIC100_qR	GACCATCAGCCCAAATCTCTC
TIC56_qF	ACCGGATTACAGGCACACTC
TIC56_qR	CTCTCCCACCATTTCTGCTCA
TIC110_qF	CTTCTCCTCGGTCTCCTCTT
TIC110_qR	GCAATCCAGTGAGCTCCTTC
TIC40_qF	CATGGAGAACCCTGATGTTGC
TIC40_qR	CATTGGGTTCTCTGAGCATTCC

Supplementary Table S3: Densitometric analysis of FtsH12 protein amounts (% of wt) in 10 day- old *ftshi* seedlings (3 replicates)

Line	Genotype	Replicates	Amount of FtsH12 (%wt)
ftshi2/FTSHi2-5	HT	a	96
		b	119
		c	136
ftshi4/FTSHi4-1	HT	a	122
		b	106
		c	128
ftshi5/FTSHi5-1	HT	a	247
		b	137
		c	174
ftshi 1-1	HM	a	429
		b	219
		c	289
ftshi 4-2	HM	a	92
		b	122
		c	167

HT: heterozygote, HM: homozygote

Table S4: Chlorophyll fluorescence parameters.

genotype	Fv/Fm		NPQ	
	short day	continuous light	short day	continuous light
wt	0.79 ± 0.003	0.79 ± 0.020	0.83 ± 0.03	1.03 ± 0.06
ox12-1	0.79 ± 0.003	0.79 ± 0.023	0.74 ± 0.06	1.00 ± 0.08
ox12-2	0.78 ± 0.005	0.79 ± 0.019	0.84 ± 0.04	0.99 ± 0.10
mi12-1	0.80 ± 0.003	0.80 ± 0.010	0.81 ± 0.05	1.02 ± 0.07
mi12-2	0.80 ± 0.005	0.80 ± 0.007	0.80 ± 0.04	0.99 ± 0.07
mi12-3	0.80 ± 0.007	0.78 ± 0.020	0.80 ± 0.05	1.05 ± 0.05

Seven-weeks-old wt and *FTSH12* mutant plants grown in short day regime (8 h light / 16 h dark) or in continuous light at 150 µmol photons m^{-2} s^{-1} were dark adapted for at least 30 min before measurement, performed at room temperature with 2000 PAR. The total measuring time was 120 s, with saturating pulses (width = 800 ms); data were collected every 10 s. Fv/Fm, Maximal PSII quantum yield; NPQ, Non-photochemical Quenching. Given are the averages of three independent biological replicates, each with five plants per experiment (mean ± SE). Student´s t-test was used for statistical evaluation.

Supplementary References:

Dubreuil C, Jin X, Barajas-López JdD, Hewitt TC, Tanz SK, Dobrenel T, Schröder WP, Hanson J, Pesquet E, Grönlund A, Small I, Strand Å. 2018. Establishment of photosynthesis through chloroplast development is controlled by two distinct regulatory phases. Plant Physiology **176**, 1199-1214.

Winter D, Vinegar B, Nahal H, Ammar R, Wilson VG, Provart N. 2007 An "Electronic fluorescent pictograph" browser for exploring and analyzing large-scale biological data sets. PLOS ONE **2**, e718
